# Unveiling Microbial Dynamics: How Forest Aging Shapes the Microbial Communities of *Pinus massoniana*

**DOI:** 10.1002/ece3.71132

**Published:** 2025-03-11

**Authors:** Guiyun Yuan, Yang Zheng, Xueguang Sun

**Affiliations:** ^1^ Institute for Forest Resources and Environment of Guizhou Guizhou University Guiyang China; ^2^ Key Laboratory of Forest Cultivation in Plateau Mountain of Guizhou Province Guizhou University Guiyang Guizhou China; ^3^ College of Forestry Guizhou University Guiyang China

**Keywords:** microbial diversity, microbial stability, *Pinus massoniana*, stand age, succession patterns

## Abstract

Plants host diverse microbial communities essential for nutrient acquisition, growth, and responses to biotic and abiotic stresses. Despite their importance, the variation and stability of these communities during forest succession remain poorly understood. This study investigated the microbial communities in *Pinus massoniana* forests at different stand ages (12, 22, 30, and 40 years). Results showed that the phyllosphere and roots of *P. massoniana* harbor diverse microbial communities, which shift dynamically with forest aging. Bacterial species diversity consistently surpassed fungal diversity across all habitats. Forest aging significantly influenced the alpha diversity of phyllosphere and soil microbes, whereas root‐associated microbial diversity remained stable. Co‐occurrence network analysis revealed that bacterial communities formed more complex networks than fungal communities and exhibited greater stability. Functional annotation confirmed that bacterial communities were functionally more stable, predominantly involving metabolic processes. In contrast, endophytes dominated the phyllosphere fungi, while ectomycorrhizal fungi were prevalent in root and soil fungal communities. Environmental factors, including total nitrogen, total phosphorus, available potassium, and pH, emerged as key drivers of microbial dynamics. These findings provide novel insights into the differing responses of bacterial and fungal communities to forest aging, highlighting the critical role of ecological niches in shaping microbial dynamics.

## Graphical abstract

Both the phyllosphere and root of *Pinus massoniana* host diverse microbes. *Pinus massoniana* forest aging induces dynamic changes in its microbiomes. The influence of forest aging on the phyllosphere microbiome overwhelms that of the root.

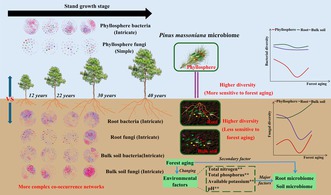

## 1 Introduction

Microorganisms inhabit both the above‐ground (phyllosphere) and below‐ground (root) parts of plants, collectively forming the plant microbiome, which plays essential roles in nutrient acquisition, growth, and responses to biotic and abiotic stress (Paasch et al. 2023; Trivedi et al. 2020; Paasch and He 2021; Sohrabi et al. 2023). In turn, plants provide these microorganisms with habitats and a steady supply of energy and carbon sources (Peiffer et al. 2013; Philippot et al. 2013; Huang et al. 2020). Given their vital ecological functions, plant‐associated microorganisms are increasingly recognized as valuable resources that can improve the cultivation of both crops and trees.

The composition of plant microbiomes is highly species‐specific and varies significantly across plant types (Laforest‐Lapointe et al. 2016). From an applied perspective, understanding the composition of a plant's microbiome is the first step toward developing management strategies that encourage a healthy microbiome favoring plant health and function. Recent studies have focused on plant‐associated microorganisms, particularly in economically important species such as wheat (Lu et al. 2024; Zhou et al. 2024; Ma et al. 2025), rice (Wang et al. 2023; Li et al. 2025), tobacco (Ren et al. 2022; Xuan et al. 2024), tea (Zhang et al. 2022; Xin et al. 2024), and apple (Cao et al. 2023, 2024). However, most of these studies have focused on microbial communities in the root and soil, with limited attention given to the phyllosphere (Shao et al. 2024). Additionally, bacteria and fungi are often studied separately (Zhang et al. 2021; Ding et al. 2023), despite both being indispensable components of the plant microbiome. Thus, a holistic approach that includes both bacterial and fungal communities across both root and phyllosphere is essential. There is also a need for more research on less‐studied tree species, particularly endemic ones, to gain deeper insights into the diversity and functionality of plant microbiomes.

Unlike herbaceous plants, forest trees have long lifespans and extended growth periods, during which physiological and environmental changes can directly or indirectly influence their microbiomes. Studies on forest trees have examined shifts in microbial communities across different stand ages (Bonet et al. 2004; Wallander et al. 2010; Zhu et al. 2010; Wang et al. 2012). For example, microbial accumulation has been reported in the soils of *Cunninghamia lanceolata* forests (Wang et al. 2018), while root‐associated microbial communities of *Hevea brasiliensis* (Herrmann et al. 2016) and *Robinia pseudoacacia* (Sheng et al. 2017) showed minimal changes. However, little attention has been paid to dynamic changes in phyllosphere microbial communities. Furthermore, most studies have focused on specific ecological niches or microbial groups, often neglecting an integrated analysis of interactions across niches. The co‐dynamics of above‐ and below‐ground microbial communities during forest aging remain poorly understood.

*Pinus massoniana* , a key afforestation species in southern China, is known for its adaptability and rapid growth, traits partially attributed to its microbiome (Sun et al. 2019; Feng et al. 2022; Feng et al. 2024). Although differences in root fungal communities across stand ages have been documented for *P. massoniana* (Dong et al. 2021), the overall response of its microbiomes to forest aging remains insufficiently explored. This study investigates microbial diversity in *P. massoniana* forests of varying stand ages through field observations. Specifically, the objectives are to (i) analyze the diversity and composition of microbial communities across different habitats (phyllosphere, roots, and soil) and (ii) examine the succession patterns of these communities during forest aging. We hypothesize that forest aging influences microbiomes in distinct ecological niches differently, with more pronounced effects on phyllosphere communities compared to the relatively stable below‐ground habitats.

## 2 Material and Methods

### 2.1 Study Area and Sampling

**FIGURE 1:**
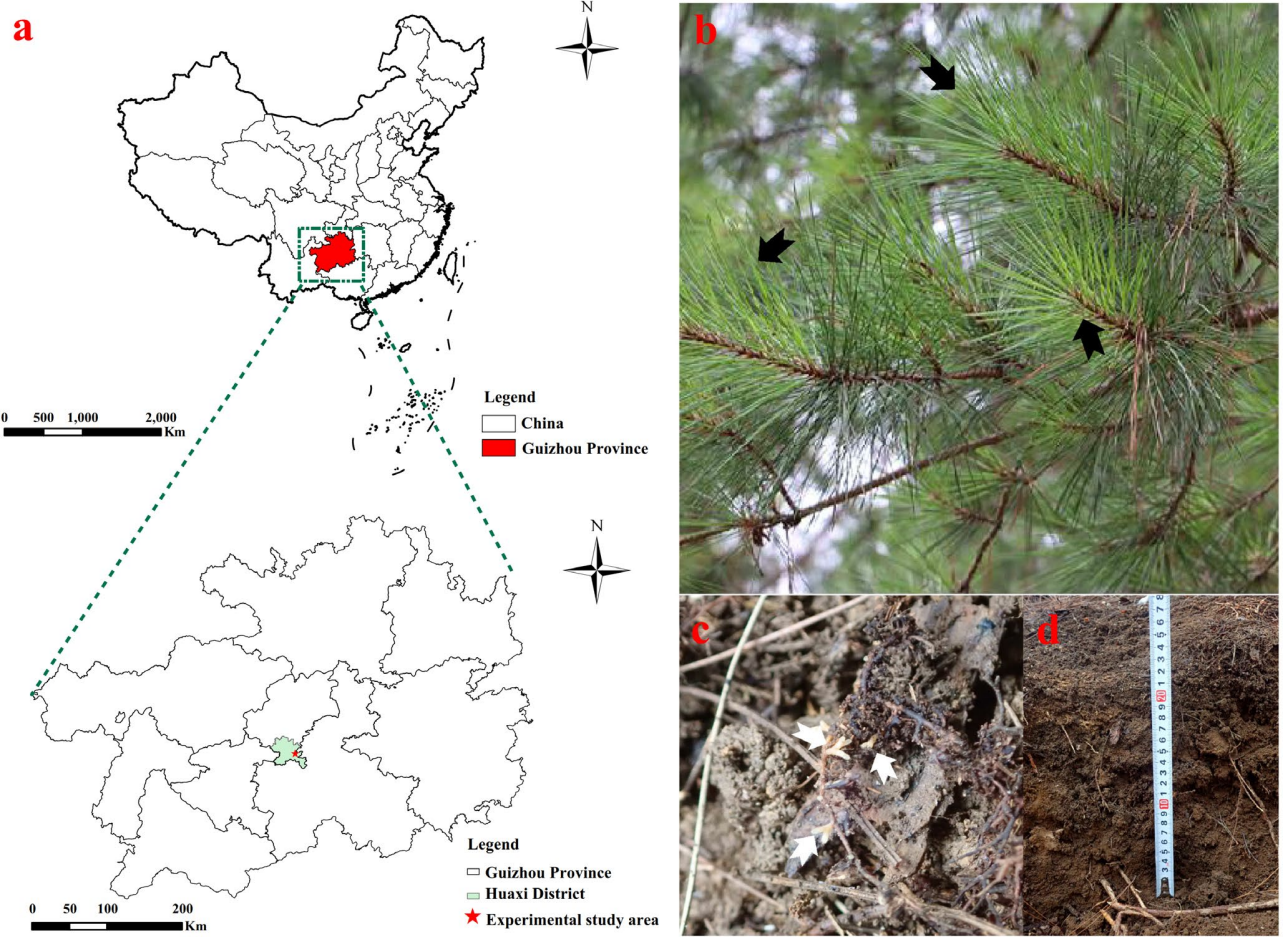
Study area and sample types. (a) Study area; (b) needle; (c) fine root; (d) bulk soil.

This study was conducted at the Mengguan state‐owned forest farm in Huaxi District, Guiyang, Guizhou Province, China (26°21′59″ N, 106°44′38″ E; altitude: 1140 m above sea level) (Figure 1a). The region experiences a subtropical monsoon climate, with an average annual temperature of 14.9°C and mean annual precipitation of 1142 mm.

In November 2022, samples were collected from four pure stands of *P. massoniana* at different ages (12, 22, 30, and 40 years). For each stand, five 20 × 20 m sampling plots were established, selected based on similar environmental characteristics, such as altitude, slope, and aspect. Within each plot, healthy mature needles were collected using high‐branch shears from three randomly selected trees, with a minimum distance of 10 m between them (Figure 1b). The needles were pooled equally to create composite samples, resulting in five composite needle samples for each stand. Fine roots (less than 2 mm in diameter) were collected from the surface soil layer (0–20 cm) beneath the canopy projection of the selected trees. Bulk soil samples were collected from outside the canopy projection, to a depth of 20 cm (Figure 1c,d). In total, 20 needle, root, and soil samples were collected separately. Needle and root samples were immediately placed in sterile plastic bags, labeled, and transported to the laboratory, where they were stored at −80°C until DNA extraction. Soil samples were divided into two groups: one group was stored at −80°C for DNA extraction, and the other was air‐dried at room temperature for soil chemical properties.

### 2.2 Determination of Soil Chemical Properties

Soil pH was measured using a pH meter (PHS‐3C, China) with a soil‐to‐water ratio of 1:2.5 (Chen et al. 2012). Total potassium (TK) and available potassium (AK) were measured using flame photometry (Kataoka et al. 1991). Available phosphorus (AP) was determined using the NaHCO_3_ method (Yaseen and Malhi 2009). Total nitrogen (TN) and alkali‐hydrolyzed nitrogen (AHN) were quantified by the semi‐micro‐Kjeldahl method (Xu et al. 2015). Total phosphorus (TP) was assessed using alkali melt digestion, followed by colorimetric analysis with the molybdenum blue method. Organic carbon content was determined using the potassium dichromate oxidation method with external heating, as described by Walkley and Black (1934).

### 2.3 DNA Extraction, Amplification, and Sequencing

DNA was extracted from soil samples using the TGuide S96 Magnetic Soil/Stool DNA Kit (model DP812) from Tiangen Biochemical Technology (Beijing) Co. Ltd. For needle and fine root samples, DNA was isolated using the CTAB method (Fu et al. 2017). DNA concentration was quantified using an enzyme labeler (Gene Company Limited, Synergistic HTX). The fungal ITS2 region was amplified using specific primer pairs ITS2F (5′‐GCATCGATGAAGAACGCAGC‐3′) and ITS2R (5′‐TCCTCCGCTTATTGATATGC‐3′) (Wen et al. 2022). The bacterial 16S V3–V4 region was amplified using the primers 338F (5′‐ACTCCTACGGGAGGCAGCA‐3′) and 806R (5′‐GGACTACHVGGGTWTCTAAT‐3′) (Castrillo et al. 2017). All PCR reactions were carried out using a Phusion High‐Fidelity PCR Master Mix (New England Biolabs). After electrophoresis on a 1.8% agarose gel, clear PCR products around 500 bp were selected for further analysis. PCR products were purified using a Monarch DNA Gel Extraction Kit (NEB, USA). Library quality was assessed using the Qsep‐400 method before high‐throughput sequencing on an Illumina NovaSeq 6000 platform, outsourced to Beijing Biomarker Biotechnology Co.

Raw sequencing reads were quality‐filtered using Trimmomatic v0.33 software (Bolger et al. 2014). Primer sequences were identified and removed using Cutadapt 1.9.1 (Martin 2011), resulting in clean reads free of primer artifacts. Amplicon sequence variants (ASVs) were generated by denoising, double‐ended sequence splicing, and removing chimeric sequences using the DADA2 method (Callahan et al. 2016) in QIIME2 2020.6 (Bolyen et al. 2019). To adjust for sampling depth, all samples were randomly resampled to match the smallest number of sequences (bacteria: 29,030; fungi: 56,575). ASVs were filtered using a threshold of 0.005% of the total number of sequences. All raw sequence data have been deposited in the NCBI Sequence Read Archive (SRA) under the following accession numbers: PRJNA1053003 (phyllosphere fungi), PRJNA1053254 (phyllosphere bacteria), PRJNA1050668 (root fungi), PRJNA1052594 (root bacteria), PRJNA1052992 (bulk soil fungi), and PRJNA1052997 (bulk soil bacteria).

### 2.4 Statistical Analysis

Statistical analyses were performed using SPSS software and R (version 4.3.1). Data normality was assessed using Shapiro–Wilk tests and histograms (*p* < 0.05). Differences in soil nutrients and microbial diversity, abundance, and stability among forest ages were evaluated using one‐way analysis of variance (ANOVA), followed by Duncan's multiple range test (*α* = 0.05). Microbial alpha and beta diversity (calculated using Bray–Curtis dissimilarity) were assessed using the “vegan” and “picante” R packages (Liu et al. 2024). Bacterial and fungal community functions were predicted using PICRUSt2 (Douglas et al. 2020) and FunGuild (Nguyen et al. 2016), respectively. Principal coordinates analysis (PCoA) and permutational multifactorial analysis of variance (PERMANOVA) with 999 permutations were performed using the “vegan” package to explore differences in community composition among phyllosphere, root, and soil samples across forest ages, based on Bray–Curtis dissimilarity (Oksanen et al. 2012). Co‐occurrence networks were constructed using Spearman's correlation of ASVs with relative abundance greater than 0.1% (Chen, Chen, et al. 2023). Node and edge data were calculated using the “psych” and “Hmisc” R packages and visualized using Gephi (version 0.10.1). The topological roles of individual nodes in the network were assessed using Zi and Pi thresholds (Ling et al. 2016). Nodes were classified as module hubs (Zi > 2.5 and Pi < 0.62), network hubs (Zi > 2.5 and Pi > 0.62), connectors (Zi < 2.5 and Pi > 0.62), or peripherals (Zi < 2.5 and Pi < 0.62). Microbial community stability was evaluated by calculating the average variation degree (AVD) based on deviations from the mean of ASV relative abundance across different stand ages, with a lower AVD indicating higher microbiome stability (Xun et al. 2021).

(1) $$|a_{i}|=\frac{|x_{i}-{\bar{x}}_{i}|}{\delta_{i}}$$

(2) $$\mathrm{AVD}=\frac{\sum_{i=1}^{n}\frac{|x_{i}-{\bar{x}}_{i}|}{\delta_{i}}}{k*n}$$

Where $a_{i}$ is the variation degree for an ASV, $x_{i}$ is the rarefied abundance of the ASV in one sample, ${\bar{x}}_{i}$ is the average rarefied abundance of the ASV in one sample group, $\delta_{i}$ is the standard deviation of the rarefied abundances of the ASV in one sample group, *k* is the number of samples in one sample group, and *n* is the number of ASVs in each sample group.

## 3 Results

### 3.1 Diversity of Microbial Communities Across Different Forest Ages

**FIGURE 2:**
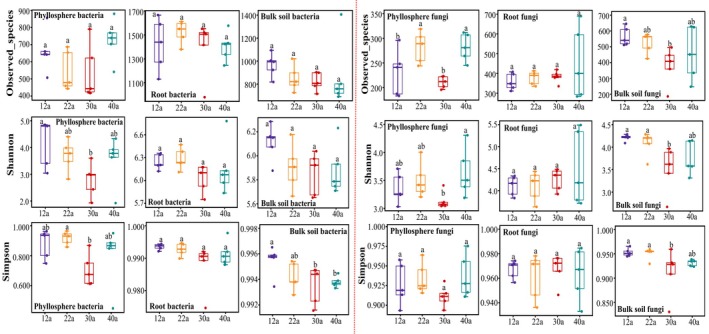
Alpha diversity index of microbial communities at different ages of *P. massoniana* forest. Different letters on the box plot represent significant differences in microbial α‐diversity index across forest ages (*p* < 0.05).

A total of 1475 bacterial ASVs and 500 fungal ASVs were obtained from the phyllosphere, 14,596 bacterial ASVs and 2946 fungal ASVs from roots, and 9512 bacterial ASVs and 3809 fungal ASVs from bulk soil, with all ASVs classified to the genus level or lower. Microbial diversity varied significantly across forest ages in the phyllosphere and bulk soil but remained stable in root‐associated communities (Figure 2). Specifically, the Observed_species and Shannon indices for phyllosphere and bulk soil microorganisms varied with forest age, while root‐associated microorganisms showed no such variation.

**FIGURE 3:**
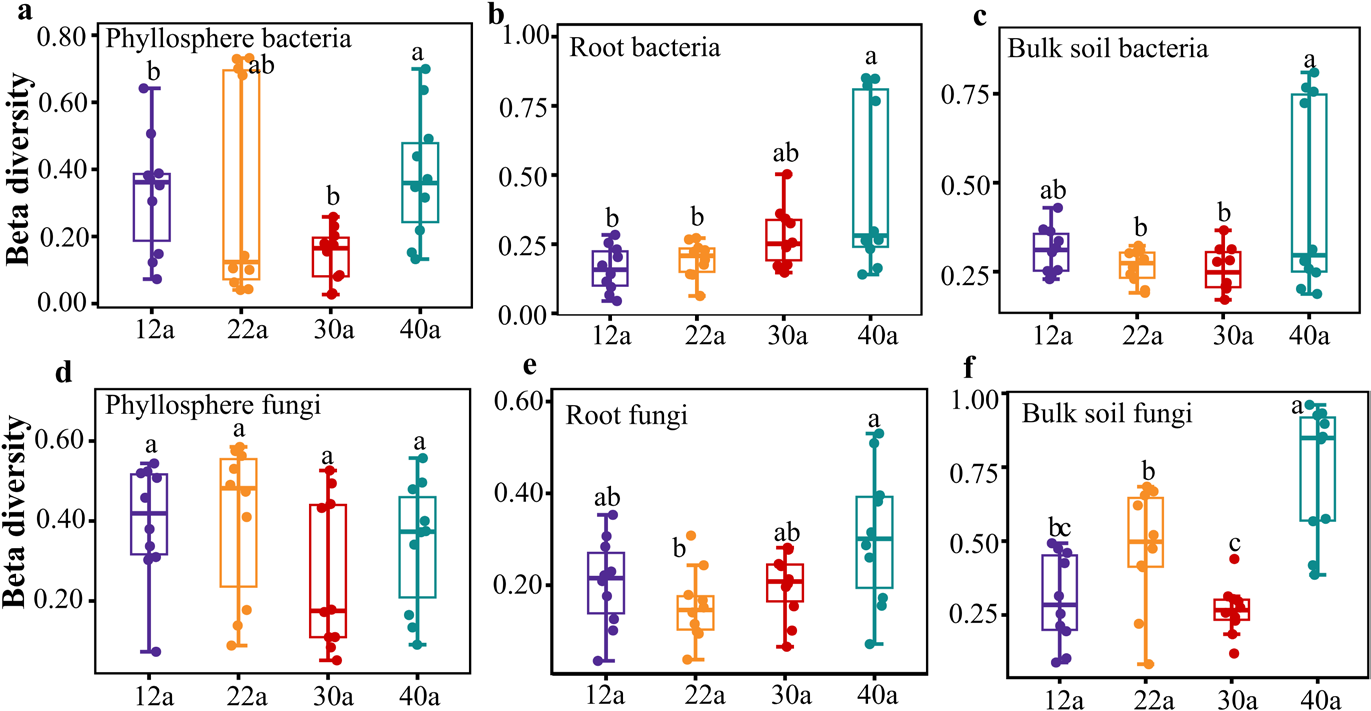
Beta diversity of microbial communities at different forest ages. Bray–Curtis dissimilarity index was used to express beta diversity. Different letters represent significant differences in microbial beta diversity index across forest ages (*p* < 0.05). Panels a, b and c show the beta diversity of phyllosphere, root, and soil bacteria, respectively. Panels d, e and f show the beta diversity of phyllosphere, root, and soil fungi, respectively.

PCoA analysis revealed significant differences in bacterial and fungal community composition across forest ages (*p* < 0.05), with the largest variation observed at 12 years (Figure S1a–f). Ecological niche compartments exerted a greater influence on community structures than forest aging (Figure S1g,h). Beta diversity analysis showed that microbial communities in the phyllosphere and bulk soil varied significantly with forest aging (Figure 3). Notably, bacterial beta diversity in the phyllosphere increased between 30 and 40 years (Figure 3a), while fungal diversity remained unaffected (Figure 3d). Root microbial communities were less impacted by forest age, exhibiting the lowest beta diversity at 22 years (Figure 3b,e). In bulk soil, both bacterial and fungal beta diversity increased at 40 years (Figure 3c,f).

### 3.2 Composition and Functional Prediction of Microbial Communities Across Forest Ages

**FIGURE 4:**
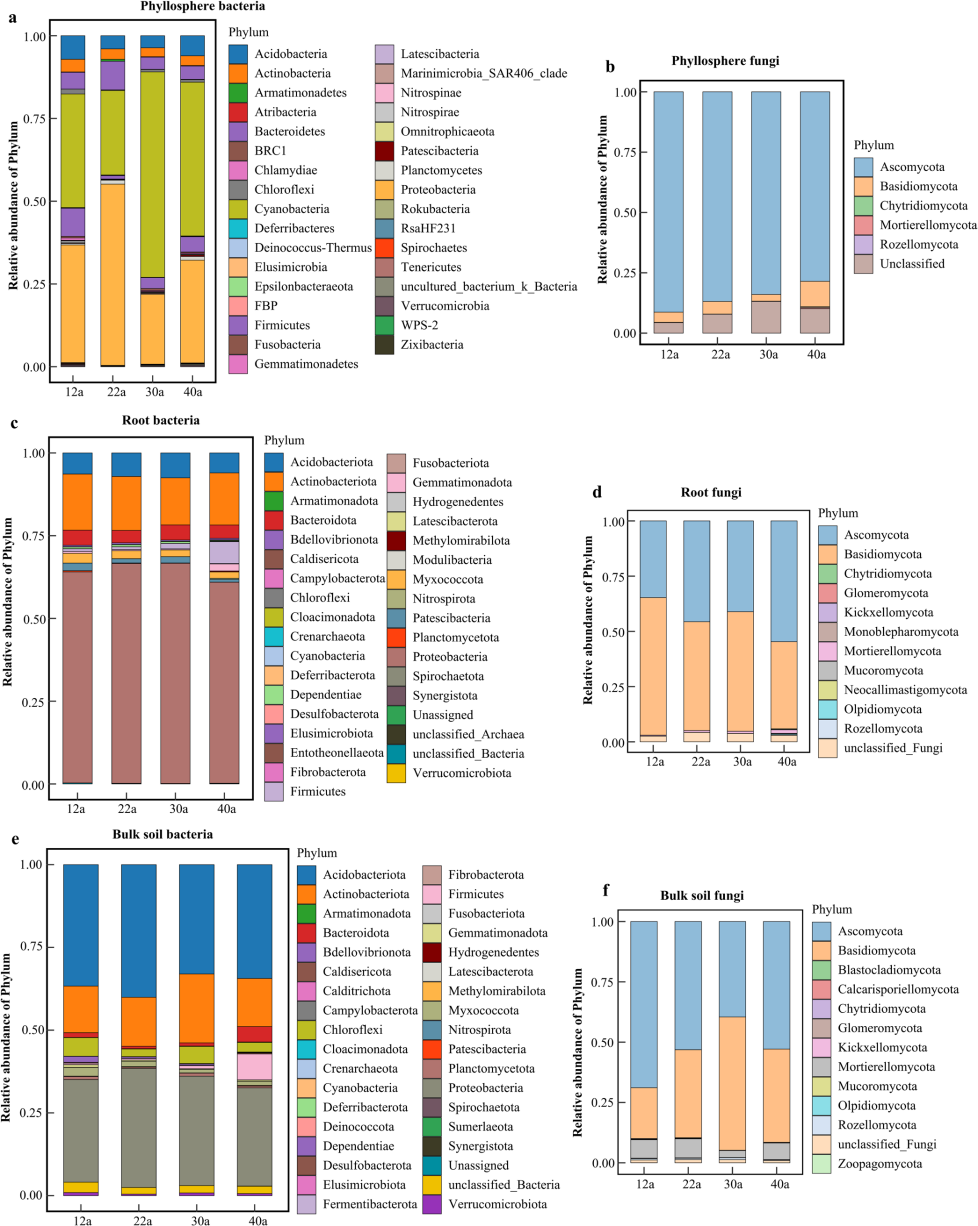
Community composition and changes of bacteria and fungi in the phyllosphere, root, and bulk soil of *P. massoniana* at different stand ages. Panels a, c and e show the community composition of phyllosphere, root, and soil bacteria, respectively. Panels b, d and f show the community composition of phyllosphere, root, and soil fungi, respectively.

**FIGURE 5:**
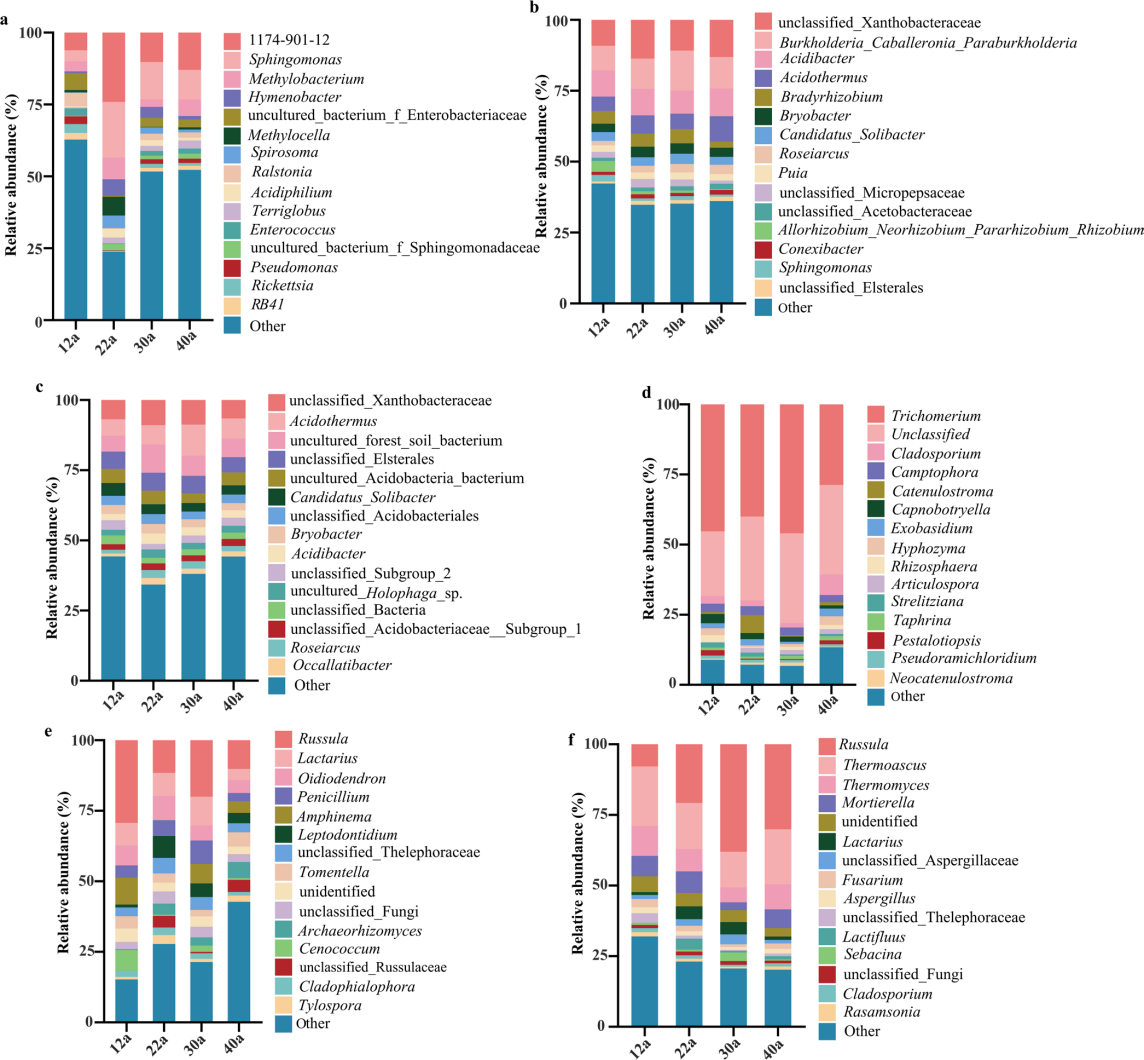
Distribution of microbial communities at the genus level across different stand ages. (a–c) show the dominant genera of phyllosphere, root, and soil bacteria, respectively. (d–f) show the dominant genera of phyllosphere, root, and soil fungi, respectively.

Microbial composition varied with forest age (Figure 4), with the dominant groups remaining relatively stable across ages. Bacterial communities in roots and bulk soil were predominantly composed of Proteobacteria, Actinobacteria, and Acidobacteria (Figure 4c,e), while Cyanobacteria, Proteobacteria, and Actinobacteria dominated the phyllosphere (Figure 4a). Fungal communities in both root and bulk soil were dominated by Ascomycota and Basidiomycota at all stand ages (Figure 4d,f), while Ascomycota was the dominant group in the phyllosphere (Figure 4b). Among the phyllosphere bacteria, the dominant genera were *1174‐901‐12*, *Sphingomonas*, and *Methylobacterium* (Figure 5a), while in roots, *Acidibacter*, *Burkholderia_Caballeronia_Paraburkholderia*, and *unclassified_Xanthobacteraceae* dominated (Figure 5b). In bulk soil, the dominant genera were *unclassified_Xanthobacteraceae*, *Acidothermus*, and *uncultured_forest_soil_bacterium* (Figure 5c). Phyllosphere fungi were mainly represented by *Trichomerium*, *Cladosporium*, and *Camptophora* (Figure 5d), while root fungi were dominated by *Russula*, *Lactarius*, and *Oidiodendron* (Figure 5e). Bulk soil fungi were primarily *Russula*, *Thermomyces*, and *Thermoascus* (Figure 5f).

**FIGURE 6:**
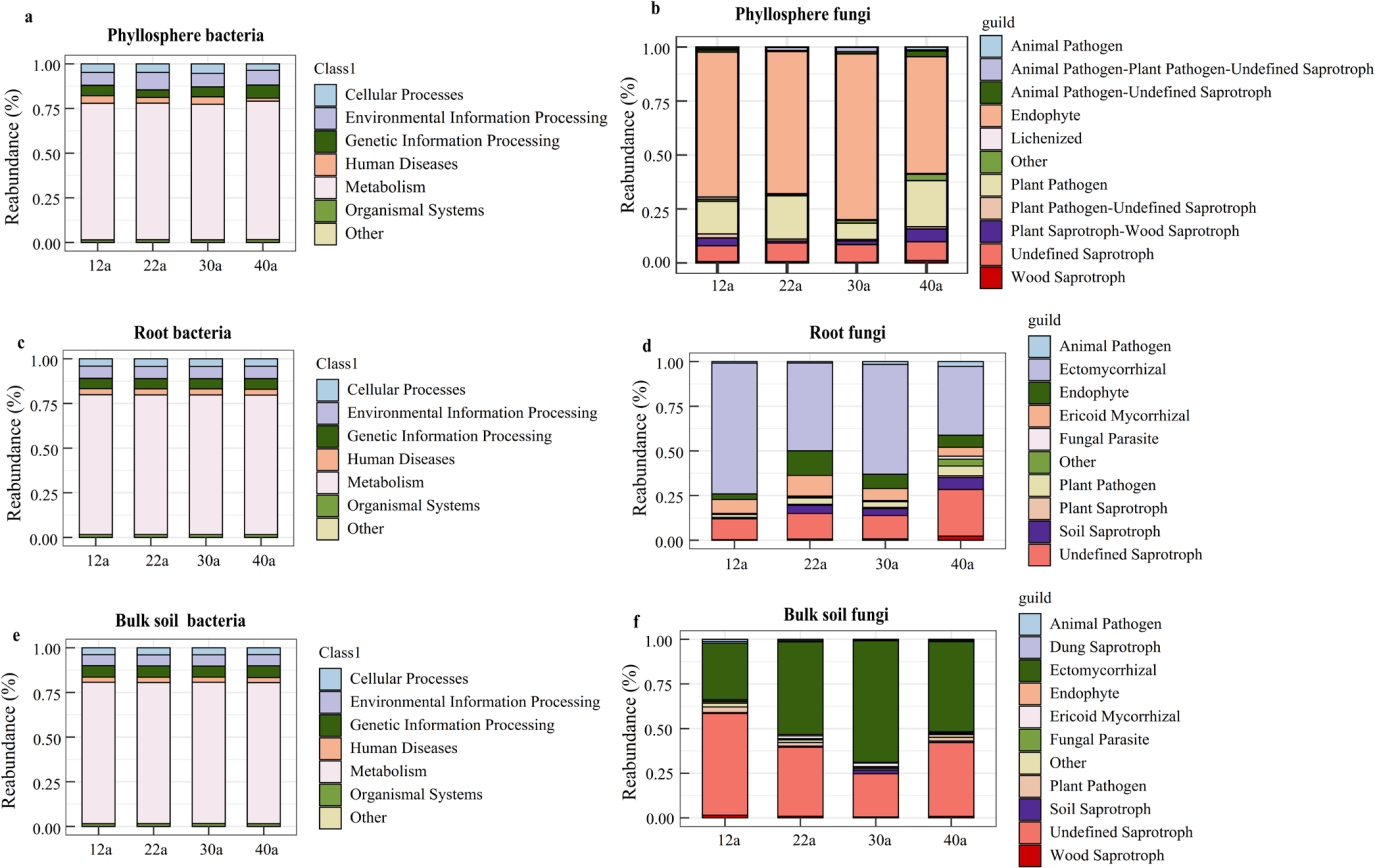
Functional prediction of microbial communities in *P. massoniana* at different forest ages. Panels a, c and e show the functional prediction of phyllosphere, root, and soil bacteria, respectively. Panels b, d and f show the functional prediction of phyllosphere, root, and soil fungi, respectively.

Bacterial communities exhibited functional stability, whereas fungal communities changed dynamically with forest age. The bacterial communities in all niches were dominated by those bacteria that were involved in metabolism (Figure 6a,c,f). Phyllosphere fungi were mostly endophytes, with maximum functional activity observed at 30 years (Figure 6b). As forest age increased, endophytic fungi declined, while plant pathogens and undefined saprotrophs increased (Figure 6b). Root and soil fungi were predominantly ectomycorrhizal fungi (ECM) (Figure 6d,f). At 12 years, ECM fungi dominated roots, but their proportion decreased with age, replaced by saprophytes and ericoid mycorrhizal fungi (Figure 6d). In contrast, ECM fungi increased in soil with age, though their proportion at 40 years was lower than at 22 and 30 years (Figure 6f).

### 3.3 Comparison of Microbial Co‐Occurrence Network and Community Stability

**FIGURE 7:**
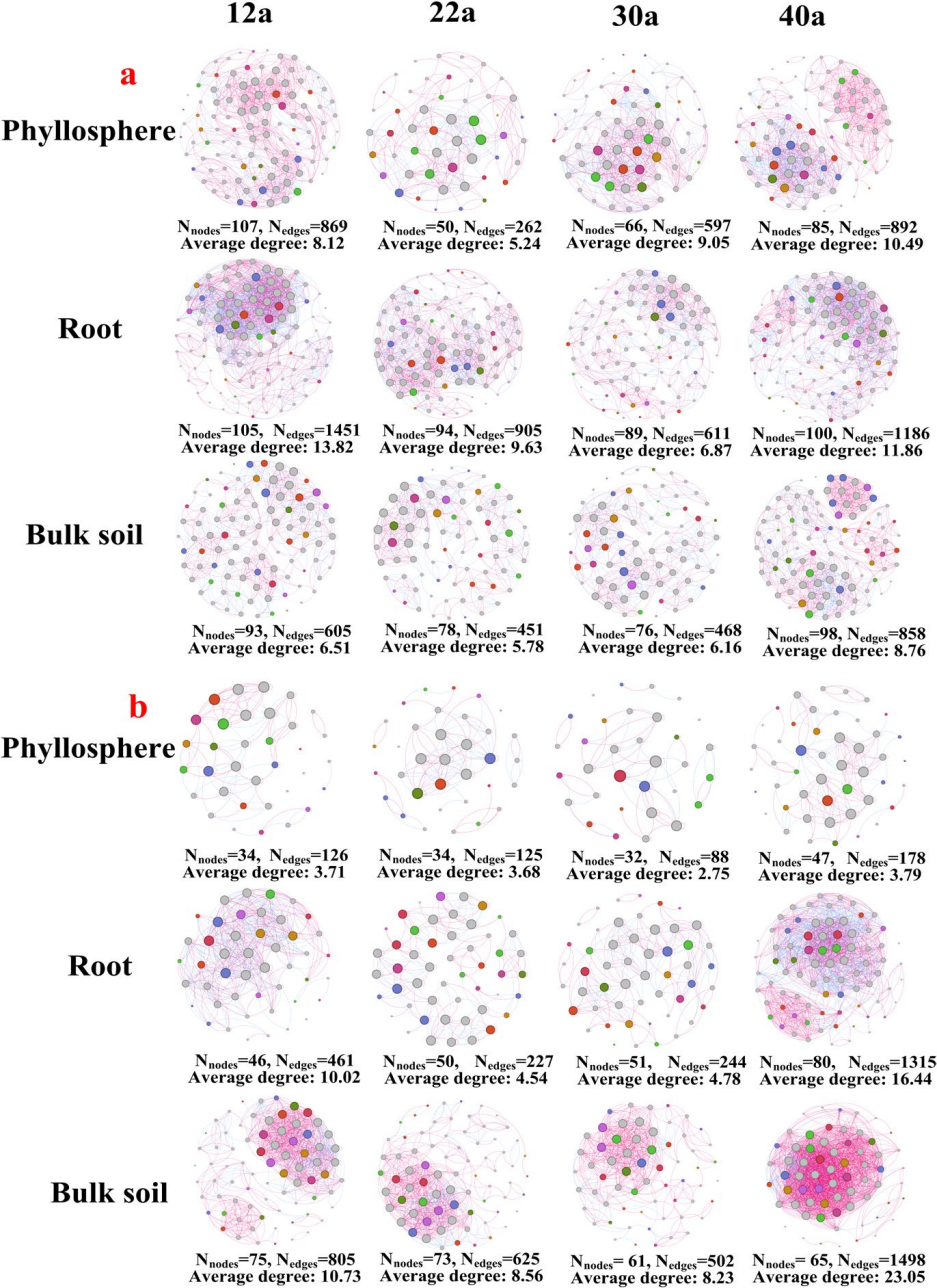
Co‐occurrence network of microbial communities in different ecological niches of *P. massoniana* . ASVs with abundance exceeding 0.1% are depicted. Positive correlations are indicated in red lines, while negative correlations are shown in blue. (a) Represents the bacterial community, and (b) represents the fungal community. Each point corresponds to a distinct genus, with points sharing identical colors denoting taxonomic classification within the same family. The point size is proportional to the relative abundance of each genus within the co‐occurrence network.

A microbial co‐occurrence network was constructed to investigate interactions within microbial communities and their responses to forest aging. Forest maturation induced dynamic changes in microbial networks, with bacterial communities forming more complex and stable networks than fungal communities (Figure 7). The phyllosphere bacterial network exhibited significantly reduced complexity at 22 years (Figure 7a), while the root bacterial network showed lower complexity at 30 years (Figure 7a). Similarly, root fungal networks demonstrated reduced complexity at 22 and 30 years. However, by 40 years, the root fungal network became more complex, characterized by increased nodes, edges, and enhanced network stability (Figure 7b). Microbial community stability, evaluated through average variation degree (AVD) values, revealed no significant differences in bacterial community stability across forest ages (Figure S2a). In contrast, fungal communities exhibited significant variation in stability with forest age (Figure S2b).

### 3.4 Relationship Between Microbial Community Diversity, Forest Age, and Soil Properties

Statistical analysis revealed significant differences in soil chemical properties, excluding organic matter, across forest ages (Table S1). A structural equation model (Figure S3) indicated that forest age had a minimal effect on the dynamic changes in microbial communities across various parts of *P. massoniana* . Instead, total nitrogen, total phosphorus, available potassium, and pH were identified as critical factors driving microbial dynamics. Notably, forest age significantly affected changes in the soil environment, which, in turn, indirectly influenced the spatial distribution and abundance of microorganisms.

## 4 Discussion

Recent studies have established that microbial compositions vary across different plant parts (Beckers et al. 2017; Wei et al. 2021), and our results confirm this pattern in *P. massoniana* . This study is novel in its demonstration for the first time of rich microbial (both bacteria and fungi) colonization in both the above‐ground (phyllosphere) and below‐ground (root) sections of *P. massoniana* , linking microbial diversity, community structure, and ecological functions to forest age. The findings expand on prior work by incorporating multiple ecological niches (phyllosphere, root, and bulk soil) and examining their relationship with forest maturation.

### 4.1 Microbial Community Diversity and Composition

Root and bulk soil microbiota harbored a more diverse array of bacterial and fungal communities than phyllosphere microbiota, supporting the previous perceptions of a rapid loss of diversity from soil to root and to shoot (Trivedi et al. 2020). We found that bacteria consistently outnumbered fungi in terms of abundance and diversity across all sampled habitats, a result that corroborates earlier studies indicating bacterial dominance in forest soils (Li et al. 2019; Schmidt et al. 2023). This dominance may be attributed to the faster growth rate and higher metabolic flexibility of bacteria compared to fungi, allowing them to adapt more readily to changes in nutrient availability and environmental conditions (Mercier and Lindow 2000).

The microbial composition across *P. massoniana* compartments exhibited both habitat specificity and successional stability. The phyllosphere's dominance of Cyanobacteria, Proteobacteria, and Actinobacteria aligns with their reported roles in nitrogen fixation (Cyanobacteria) and stress adaptation (Proteobacteria/Actinobacteria) in conifer ecosystems (Sun et al. 2021; Chen, Xiao, et al. 2023). In contrast, the predominance of Acidobacteria in the root and soil reflects its oligotrophic adaptation to acidic soils, consistent with global forest soil surveys (Sohrabi et al. 2023). Genus‐level patterns revealed functional specialization. Phyllosphere colonizers *Sphingomonas* and *Methylobacterium* likely enhance host defense via antimicrobial production and methanol metabolism, respectively (Asaf et al. 2020; Li et al. 2022), while root‐associated *Burkholderia_Caballeronia_Paraburkholderia* may promote plant growth by improving nutrient uptake (Rojas‐Rojas et al. 2025). The cellulolytic thermophile *Acidothermus* in bulk soil further indicates intensified organic decomposition in older stands (Berry et al. 2014).

Fungal communities showed compartmentalized symbiosis strategies. The co‐dominance of Ascomycota/Basidiomycota in roots and soil mirrors their complementary roles in decomposition and mycorrhizal symbiosis (Cregger et al. 2018). Specifically, ECM fungi *Russula* and *Lactarius* likely facilitate pine nutrient uptake (Hobbie et al. 2014; Tang et al. 2021), whereas the ubiquitous *Oidiodendron* suggests broader host compatibility than previously reported in mature forests (Dong et al. 2021), possibly mediated by its dual ericoid/ECM symbiosis capacity (Martino et al. 2018). The persistent phyllosphere dominance of *Trichomerium* highlights its unrecognized role in conifer stress tolerance, potentially through melanin‐mediated UV/drought resistance (Chomnunti et al. 2012).

### 4.2 Forest Aging and Changes in Microbial Diversity, Community Stability, and Function

Interestingly, while soil and phyllosphere microbial diversity exhibited significant age‐related changes, root‐associated microbial diversity remained relatively stable across stand ages. This suggests the resilience of root‐associated microbiomes to fluctuations in the broader forest environment (van der Heijden et al. 2008). This resilience may stem from long‐term symbiotic relationships between *P. massoniana* and its root microbiome, which are less susceptible to short‐term environmental changes compared to soil or canopy‐associated microbes. In contrast, the phyllosphere is exposed to varying stresses, including humidity, solar radiation, and desiccation, creating a highly dynamic and unstable environment (Vorholt 2012; Müller et al. 2016; Miura et al. 2019). These fluctuating conditions likely account for the significant influence of forest age on phyllosphere microbial communities, emphasizing the disparity between above‐ and below‐ground habitats.

Notably, the stability of microbial communities also differed between forest compartments. Generally, both the bacterial and fungal communities in the roots showed pronounced stability, which is likely due to the higher reliance of microbes on root exudates and plant debris (Sun et al. 2017; Zhong et al. 2018). However, we observed a notable shift at 40 years, with greater stability in the phyllosphere microbial community compared to root microorganisms. This pattern supports the idea that forest maturation leads to the development of more complex microbial networks, as microbial species establish niche‐based interactions that enhance ecosystem stability (Peay et al. 2016). The ECM fungal taxa of overmatured forest were linked to organic matter decomposition (Kyaschenko et al. 2017), and as saprophytic fungi increase with forest maturity, the nutrient competition between them may drive root fungal community instability (Kohler et al. 2015; Lindahl et al. 2021). This observation is novel and suggests that nutrient availability, fungal competition, and ecological niches in overmature forests influence the stability of phyllosphere and root‐associated microbes.

Functional annotation confirmed that the functional groups of fungi changed significantly with forest aging, with the relative abundances of ECM fungi being the highest in 12a, and the proportion of saprophytic fungi gradually increased as forest aging. This may be closely related to the fact that plants in the rapid growth period can allocate more photosynthetic products to fine roots, changing the scope of ECM symbiosis (Toju et al. 2014; Carriconde et al. 2019; Tedersoo and Bahram 2019). Endophytes, an important group found in all plants (Collinge et al. 2022), are many endophytes living in the phyllosphere of *P. massoniana* and play a major role in various forest ages and may be beneficial to the growth of *P. massoniana* and enhance the ability to adapt to abiotic stress and resist pests and diseases (Hardoim et al. 2015; Jørgensen et al. 2020).

Using PCoA and SEM analysis, we found that while forest age had a minor effect on microbial community structure, soil properties such as total nitrogen, total phosphorus, available potassium, and pH were key drivers of microbial dynamics. These factors have been identified in previous studies as primary determinants of microbial community structure (Yang et al. 2023; Zhang et al. 2023). Importantly, phyllosphere bacterial abundance showed a significant age‐related shift, suggesting that bacterial communities in the phyllosphere are highly sensitive to environmental changes along with tree aging (Vorholt 2012; Müller et al. 2016; Miura et al. 2019).

## 5 Conclusion

**FIGURE 8:**
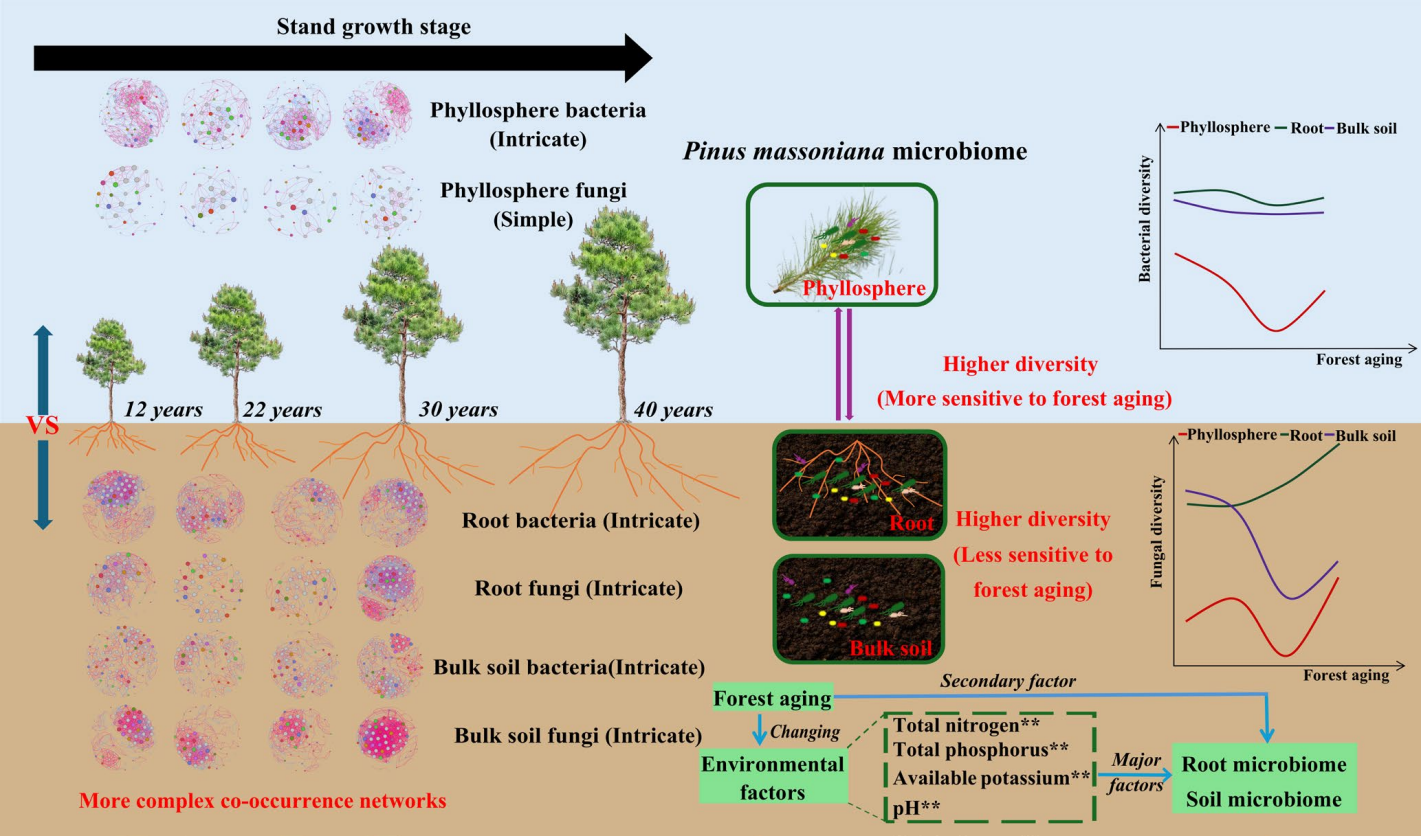
Schematic diagram illustrating the microbiome changes in *P. massoniana* with forest aging.

This study highlights the dynamic shifts in microbial community diversity and composition in *P. massoniana* forests, with forest aging influencing microbial diversity in the phyllosphere and bulk soil but having little effect on root‐associated microorganisms (Figure 8). Notably, the co‐occurrence networks between root bacteria and bulk soil fungi evolved with forest maturation, emphasizing the role of soil properties, such as nitrogen, phosphorus, available potassium, and pH, in shaping microbial dynamics. These results suggest that environmental factors, rather than forest age alone, play a more substantial role in shaping microbial dynamics. Future research should focus on elucidating the functional mechanisms underlying the interactions between microbial communities and soil properties, particularly in the context of nutrient cycling and forest ecosystem resilience. Additionally, investigating the potential application of key microbial taxa as bioinoculants could provide innovative strategies for enhancing forest productivity and sustainability in *P. massoniana* plantations.

## Author Contributions

**Guiyun Yuan:** data curation (equal), formal analysis (equal), investigation (equal), writing – original draft (equal), writing – review and editing (equal). **Yang Zheng:** data curation (equal), investigation (equal). **Xueguang Sun:** conceptualization (equal), funding acquisition (equal), methodology (equal), writing – review and editing (equal).

## Conflicts of Interest

The authors declare no conflicts of interest.

## Supporting information


Figure S1.

Table S1:

## Data Availability

The data that support the findings of this study are available in the Supporting material of this article. The raw data were archived under NCBI Sequence Read Archive (SRA) and their accession numbers are available in “Material and methods”.

## References

[ece371132-bib-0001] Asaf, S. , M. Numan , A. Khan , and A. Al‐Harrasi . 2020. “Sphingomonas: From Diversity and Genomics to Functional Role in Environmental Remediation and Plant Growth.” Critical Reviews in Biotechnology 40: 1–15. 10.1080/07388551.2019.1709793. 31906737

[ece371132-bib-0002] Beckers, B. , M. de op Beeck , N. Weyens , W. Boerjan , and J. Vangronsveld . 2017. “Structural Variability and Niche Differentiation in the Rhizosphere and Endosphere Bacterial Microbiome of Field‐Grown Poplar Trees.” Microbiome 5, no. 1: 25. 10.1186/s40168-017-0241-2. 28231859 PMC5324219

[ece371132-bib-0003] Berry, A. M. , R. D. Barabote , and P. Normand . 2014. “The Family acidothermaceae.” In The Prokaryotes, edited by E. Rosenberg , E. F. DeLong , S. Lory , E. Stackebrandt , and F. Thompson . Springer. 10.1007/978-3-642-30138-4_199.

[ece371132-bib-0004] Bolger, A. M. , M. Lohse , and B. Usadel . 2014. “Trimmomatic: a flexible trimmer for Illumina sequence data.” Bioinformatics 30: 2114–2120. 10.1093/bioinformatics/btu170. 24695404 PMC4103590

[ece371132-bib-0005] Bolyen, E. , J. R. Rideout , M. R. Dillon , et al. 2019. “Reproducible, Interactive, Scalable and Extensible Microbiome Data Science Using Qiime 2.” Nature Biotechnology 37, no. 8: 852–857. 10.1038/s41587-019-0209-9. PMC7015180 31341288

[ece371132-bib-0006] Bonet, J. A. , C. R. Fischer , and C. Colinas . 2004. “The Relationship Between Forest Age and Aspect on the Production of Sporocarps of Ectomycorrhizal fungi in *Pinus sylvestris* Forests of the Central Pyrenees.” Forest Ecology and Management 203, no. 1: 157–175. 10.1016/j.foreco.2004.07.063.

[ece371132-bib-0007] Callahan, B. J. , P. J. Mcmurdie , M. J. Rosen , A. W. Han , A. J. A. Johnson , and S. P. Holmes . 2016. “Dada2: High‐Resolution Sample Inference From Illumina Amplicon Data.” Nature Methods 13, no. 7: 581–583. 10.1038/nmeth.3869. 27214047 PMC4927377

[ece371132-bib-0008] Cao, Y. , P. Du , Z. Li , J. Xu , C. Ma , and B. Liang . 2024. “Melatonin Promotes the Recovery of Apple Plants After Waterlogging by Shaping the Structure and Function of the Rhizosphere Microbiome.” Plant, Cell & Environment 47, no. 7: 2612–2628. 10.1111/pce.14903. 38712467

[ece371132-bib-0009] Cao, Y. , P. Du , J. Zhang , J. Ji , J. Xu , and B. Liang . 2023. “Dopamine Alleviates Cadmium Stress in Apple Trees by Recruiting Beneficial Microorganisms to Enhance the Physiological Resilience Revealed by High‐Throughput Sequencing and Soil Metabolomics.” Horticulture Research 10, no. 7: uhad112. 10.1093/hr/uhad112. 37577402 PMC10419553

[ece371132-bib-0010] Carriconde, F. , M. Gardes , J.‐M. Bellanger , et al. 2019. “Host Effects in High Ectomycorrhizal Diversity Tropical Rainforests on Ultramafic Soils in New Caledonia.” Fungal Ecology 39: 201–212. 10.1016/j.funeco.2019.02.006.

[ece371132-bib-0011] Castrillo, G. , P. J. P. Lima Teixeira , S. H. Paredes , et al. 2017. “Root Microbiota Drive Direct Integration of Phosphate Stress and Immunity.” Nature 543: 513–518. 10.1038/nature21417. 28297714 PMC5364063

[ece371132-bib-0012] Chen, J. , Q. C. Xiao , D. L. Xu , et al. 2023. “Soil Microbial Community Composition and Co‐Occurrence Network Responses to Mild and Severe Disturbances in Volcanic Areas.” Science of the Total Environment 901: 165889. 10.1016/j.scitotenv.2023.165889. 37524180

[ece371132-bib-0013] Chen, L. M. , S. S. Chen , Y. Zhang , et al. 2023. “Co‐Occurrence Network of Microbial Communities Affected by Application of Anaerobic Fermentation Residues During Phytoremediation of Ionic Rare Earth Tailings Area.” Science of the Total Environment 856: 159223. 10.1016/j.scitotenv.2022.159223. 36208748

[ece371132-bib-0014] Chen, X. M. , K. Fang , and C. Chen . 2012. “Seasonal Variation and Impact Factors of Available Phosphorus in Typical Paddy Soils of Taihu Lake Region, China.” Water and Environment Journal 26, no. 3: 392–398. 10.1111/j.1747-6593.2011.00299.x.

[ece371132-bib-0015] Chomnunti, P. , D. J. Bhat , E. Jones , E. Chukeatirote , A. Bahkali , and K. Hyde . 2012. “Trichomeriaceae, a New Sooty Mould Family of Chaetothyriales.” Fungal Diversity 56, no. 1: 63–76. 10.1007/s13225-012-0197-2.

[ece371132-bib-0016] Collinge, D. B. , B. Jensen , and H. J. Jørgensen . 2022. “Fungal Endophytes in Plants and Their Relationship to Plant Disease.” Current Opinion in Microbiology 69: 102177. 10.1016/j.mib.2022.102177. 35870225

[ece371132-bib-0017] Cregger, M. A. , A. M. Veach , Z. K. Yang , et al. 2018. “The Populus Holobiont: Dissecting the Effects of Plant Niches and Genotype on the Microbiome.” Microbiome 6, no. 1: 31. 10.1186/s40168-018-0413-8. 29433554 PMC5810025

[ece371132-bib-0018] Ding, C. , X. Xu , Y. Liu , et al. 2023. “Diversity and Assembly of Active bacteria and Their Potential Function Along Soil Aggregates in a Paddy Field.” Science of the Total Environment 866: 161360. 10.1016/j.scitotenv.2022.161360. 36610629

[ece371132-bib-0019] Dong, H. Y. , J. F. Ge , K. Sun , et al. 2021. “Change in Root‐Associated Fungal Communities Affects Soil Enzymatic Activities During *Pinus massoniana* Forest Development in Subtropical China.” Forest Ecology and Management 482: 118817. 10.1016/j.foreco.2020.118817.

[ece371132-bib-0020] Douglas, G. M. , V. J. Maffei , J. Zaneveld , et al. 2020. “PICRUSt2 for prediction of metagenome functions.” Nature Biotechnology 38, no. 6: 685–688. 10.1038/s41587-020-0548-6. PMC7365738 32483366

[ece371132-bib-0021] Feng, W. Y. , J. F. Feng , G. J. Ding , and X. G. Sun . 2024. “Metabolic Adaptations of *Pinus massoniana* to Low Phosphorus and Acidic Aluminium With or Without Ectomycorrhization.” Environmental and Experimental Botany 218: 105619. 10.1016/j.envexpbot.2023.105619.

[ece371132-bib-0022] Feng, Y. H. , T. F. Shen , Z. Q. Yang , et al. 2022. “Identification of Genes Involved in Oleoresin Biosynthesis in *Pinus massoniana* Through the Combination of SMRT and Illumina Sequencing.” Industrial Crops and Products 188: 115553. 10.1016/j.indcrop.2022.115553.

[ece371132-bib-0023] Fu, Z. , J. Song , and P. E. Jameson . 2017. “A Rapid and Cost Effective Protocol for Plant Genomic DNA Isolation Using Regenerated Silica Columns in Combination With CTAB Extraction.” Journal of Integrative Agriculture 16, no. 8: 1682–1688. 10.1016/S2095-3119(16)61534-4.

[ece371132-bib-0024] Hardoim, P. R. , L. S. van Overbeek , G. Berg , et al. 2015. “The Hidden World Within Plants: Ecological and Evolutionary Considerations for Defining Functioning of Microbial Endophytes.” Microbiology and Molecular Biology Reviews 79, no. 3: 293–320. 10.1128/mmbr.00050-14. 26136581 PMC4488371

[ece371132-bib-0025] Herrmann, L. , D. Lesueur , L. Bräu , et al. 2016. “Diversity of Root‐Associated Arbuscular Mycorrhizal Fungal Communities in a Rubber Tree Plantation Chronosequence in Northeast Thailand.” Mycorrhiza 26: 863–877. 10.1007/s00572-016-0720-5. 27448680

[ece371132-bib-0026] Hobbie, E. A. , L. T. A. van Diepen , E. A. Lilleskov , A. P. Ouimette , A. C. Finzi , and K. S. Hofmockel . 2014. “Fungal Functioning in a Pine Forest: Evidence From a 15N‐Labeled Global Change Experiment.” New Phytologist 201: 1431–1439. 10.1111/nph.12578. 24304469

[ece371132-bib-0027] Huang, R. , P. Chen , X. Wang , et al. 2020. “Structural Variability and Niche Differentiation of the Rhizosphere and Endosphere Fungal Microbiome of *Casuarina equisetifolia* at Different Ages.” Brazilian Journal of Microbiology 51, no. 4: 1873–1884. 10.1007/s42770-020-00337-7. 32661898 PMC7688850

[ece371132-bib-0028] Jørgensen, H. J. L. , D. B. Collinge , E. C. Rojas , et al. 2020. Plant Endophytes. John Wiley & Sons, Ltd. 10.1002/9780470015902.a0028893.

[ece371132-bib-0029] Kataoka, H. , Y. Ueno , and M. Makita . 1991. “Analysis of O‐Phosphoamino Acids in Proteins by Gas Chromatography With Flame Photometric Detection.” Agricultural and Biological Chemistry 55, no. 6: 1587–1592. 10.1080/00021369.1991.10870792.

[ece371132-bib-0030] Kohler, A. , A. Kuo , L. G. Nagy , et al. 2015. “Convergent Losses of Decay Mechanisms and Rapid Turnover of Symbiosis Genes in Mycorrhizal Mutualists.” Nature Genetics 47, no. 4: 410–415. 10.1038/ng.3223. 25706625

[ece371132-bib-0031] Kyaschenko, J. , K. E. Clemmensen , A. Hagenbo , E. Karltun , and B. D. Lindahl . 2017. “Shift in Fungal Communities and Associated Enzyme Activities Along an Age Gradient of Managed *Pinus sylvestris* Stands.” ISME Journal 11, no. 4: 863–874. 10.1038/ismej.2016.184. 28085155 PMC5364365

[ece371132-bib-0032] Laforest‐Lapointe, I. , C. Messier , and S. W. Kembel . 2016. “Host Species Identity, Site and Time Drive Temperate Tree Phyllosphere Bacterial Community Structure.” Microbiome 4: 27. 10.1186/s40168-016-0174-1. 27316353 PMC4912770

[ece371132-bib-0033] Li, J. , H.‐W. Hu , Z.‐J. Cai , et al. 2019. “Contrasting Soil Bacterial and Fungal Communities Between the Swamp and Upland in the Boreal Forest and Their Biogeographic Distribution Patterns.” Wetlands 9, no. 3: 441–451. 10.1007/s13157-018-1086-6.

[ece371132-bib-0034] Li, P. D. , Z. R. Zhu , Y. Z. Zhang , et al. 2022. “The Phyllosphere Microbiome Shifts Toward Combating Melanose Pathogen.” Microbiome 10, no. 1: 56. 10.1186/s40168-022-01234-x. 35366955 PMC8976405

[ece371132-bib-0035] Li, Q. , Y. Liu , N. Su , et al. 2025. “Knowledge‐Based Phosphorus Input Levels Control the Link Between Soil Microbial Diversity and Ecosystem Functions in Paddy Fields.” Agriculture, Ecosystems & Environment 379: 109352. 10.1016/j.agee.2024.109352.

[ece371132-bib-0036] Lindahl, B. D. , J. Kyaschenko , K. Varenius , et al. 2021. “A Group of Ectomycorrhizal fungi Restricts Organic Matter Accumulation in Boreal Forest.” Ecology Letters 24, no. 7: 1341–1351. 10.1111/ele.13746. 33934481

[ece371132-bib-0037] Ling, N. , C. Zhu , C. Xue , et al. 2016. “Insight Into How Organic Amendments Can Shape the Soil Microbiome in Long‐Term Field Experiments as Revealed by Network Analysis.” Soil Biology and Biochemistry 99: 137–149. 10.1016/j.soilbio.2016.05.005.

[ece371132-bib-0038] Liu, M. , X. G. Lv , W. G. Zhang , et al. 2024. “Biological Interactions Control Bacterial but Not Fungal β Diversity During Vegetation Degradation in Saline‐Alkaline Soil.” Science of the Total Environment 919: 170826. 10.1016/j.scitotenv.2024.170826. 38340840

[ece371132-bib-0039] Lu, J. , X. Yin , K. Qiu , et al. 2024. “Wheat Cultivar Replacement Drives Soil Microbiome and Microbial Cooccurrence Patterns.” Agriculture, Ecosystems & Environment 360: 108774. 10.1016/j.agee.2023.108774.

[ece371132-bib-0040] Ma, L. , J. Zhang , H. Li , et al. 2025. “Key Microbes in Wheat Maize Rotation Present Better Promoting Wheat Yield Effect in a Variety of Crop Rotation Systems.” Agriculture, Ecosystems & Environment 379: 109370. 10.1016/j.agee.2024.109370.

[ece371132-bib-0041] Martin, M. 2011. “Cutadapt Removes Adapter Sequences From High‐Throughput Sequencing Reads.” EMBnet.Journal 17: 10–12. 10.14806/ej.17.1.200.

[ece371132-bib-0042] Martino, E. , E. Morin , G.‐A. Grelet , et al. 2018. “Comparative Genomics and Transcriptomics Depict Ericoid Mycorrhizal fungi as Versatile Saprotrophs and Plant Mutualists.” New Phytologist 217, no. 3: 1213–1229. 10.1111/nph.14974. 29315638

[ece371132-bib-0043] Mercier, J. , and S. E. Lindow . 2000. “Role of Leaf Surface Sugars in Colonization of Plants by Bacterial Epiphytes.” Applied and Environmental Microbiology 66: 369–374. 10.1128/AEM.66.1.369-374.2000. 10618250 PMC91832

[ece371132-bib-0044] Miura, T. , R. Sánchez , L. E. Castañeda , K. Godoy , and O. Barbosa . 2019. “Shared and Unique Features of Bacterial Communities in Native Forest and Vineyard Phyllosphere.” Ecology and Evolution 9, no. 6: 3295–3305. 10.1002/ece3.4949. 30962893 PMC6434556

[ece371132-bib-0045] Müller, D. B. , C. Vogel , Y. Bai , and J. A. Vorholt . 2016. “The Plant Microbiota: Systems‐Level Insights and Perspectives.” Annual Review of Genetics 50, no. 1: 211–234. 10.1146/annurev-genet-120215-034952. 27648643

[ece371132-bib-0046] Nguyen, N. H. , Z. Song , S. T. Bates , et al. 2016. “Funguild: An Open Annotation Tool for Parsing Fungal Community Datasets by Ecological Guild.” Fungal Ecology 20: 241–248. 10.1016/j.funeco.2015.06.006.

[ece371132-bib-0047] Oksanen, J. , B. Guillaume , M. Friendly , et al. 2012. Vegan: Community Ecology Package. Ordination Methods. Diversity Analysis and Other Functions for Community and Vegetation Ecologists. R package version 2.5‐7.

[ece371132-bib-0048] Paasch, B. C. , and S. Y. He . 2021. “Toward Understanding Microbiota Homeostasis in the Plant Kingdom.” PLoS Pathogens 17, no. 4: e1009472. 10.1371/journal.ppat.1009472. 33886694 PMC8061798

[ece371132-bib-0049] Paasch, B. C. , R. Sohrabi , J. M. Kremer , et al. 2023. “A Critical Role of a Eubiotic Microbiota in Gating Proper Immunocompetence in Arabidopsis.” Nature Plants 9: 1468–1480. 10.1038/s41477-023-01501-1. 37591928 PMC10505558

[ece371132-bib-0050] Peay, K. G. , P. G. Kennedy , and J. M. Talbot . 2016. “Dimensions of Biodiversity in the Earth Mycobiome.” Nature Reviews. Microbiology 14, no. 7: 434–447. 10.1038/nrmicro.2016.59. 27296482

[ece371132-bib-0051] Peiffer, J. A. , A. Spor , O. Koren , et al. 2013. “Diversity and Heritability of the Maize Rhizosphere Microbiome Under Field Conditions.” Proceedings of the National Academy of Sciences 110: 6548–6553. 10.1073/pnas.1302837110. PMC3631645 23576752

[ece371132-bib-0052] Philippot, L. , J. M. Raaijmakers , P. Lemanceau , and W. H. van der Putten . 2013. “Going Back to the Roots: The Microbial Ecology of the Rhizosphere.” Nature Reviews. Microbiology 11, no. 11: 789–799. 10.1038/nrmicro3109. 24056930

[ece371132-bib-0053] Ren, T. , H. Feng , C. Xu , et al. 2022. “Exogenous Application and Interaction of Biochar With Environmental Factors for Improving Functional Diversity of rhizosphere's Microbial Community and Health.” Chemosphere 294: 133710. 10.1016/j.chemosphere.2022.133710. 35074326

[ece371132-bib-0054] Rojas‐Rojas, F. U. , I. M. Gómez‐Vázquez , P. de Estrada‐ los Santos , H. Shimada‐Beltrán , and J. C. Vega‐Arreguín . 2025. “The Potential of Paraburkholderia Species to Enhance Crop Growth.” World Journal of Microbiology and Biotechnology 41: 62. 10.1007/s11274-025-04256-3. 39904926 PMC11794353

[ece371132-bib-0055] Schmidt, J. E. , A. S. Puig , A. E. Duval , E. E. Pfeufer , and S. G. Tringe . 2023. “Phyllosphere Microbial Diversity and Specific Taxa Mediate Within‐Cultivar Resistance to Phytophthora Palmivora in Cacao.” mSphere 8, no. 5: e00013‐23. 10.1128/msphere.00013-23. 37603690 PMC10597403

[ece371132-bib-0056] Shao, Q. , Q. Ran , X. Li , C. Dong , J. Huang , and Y. Han . 2024. “Deciphering the Effect of Phytohormones on the Phyllosphere Microbiota of *Eucommia ulmoides* .” Microbiological Research 278: 127513. 10.1016/j.micres.2023.127513. 37837828

[ece371132-bib-0057] Sheng, M. , X. D. Chen , X. L. Zhang , et al. 2017. “Changes in Arbuscular Mycorrhizal Fungal Attributes Along a Chronosequence of Black Locust (*Robinia pseudoacacia*) Plantations Can Be Attributed to the Plantation‐Induced Variation in Soil Properties.” Science of the Total Environment 599‐600: 273–283. 10.1016/j.scitotenv.2017.04.199. 28477484

[ece371132-bib-0058] Sohrabi, R. , B. C. Paasch , J. A. Liber , and S. Y. He . 2023. “Phyllosphere Microbiome.” Annual Review of Plant Biology 74, no. 1: 539–568. 10.1146/annurev-arplant-102820-032704. 36854478

[ece371132-bib-0059] Sun, A. Q. , X. Y. Jiao , Q. L. Chen , et al. 2021. “Microbial Communities in Crop Phyllosphere and Root Endosphere Are More Resistant Than Soil Microbiota to Fertilization.” Soil Biology and Biochemistry 153: 108113. 10.1016/j.soilbio.2020.108113.

[ece371132-bib-0060] Sun, S. , S. Li , B. N. Avera , B. D. Strahm , and B. D. Badgley . 2017. “Soil Bacterial and Fungal Communities Show Distinct Recovery Patterns During Forest Ecosystem Restoration.” Applied and Environmental Microbiology 83, no. 14: e00966‐17. 10.1128/AEM.00966-17. 28476769 PMC5494632

[ece371132-bib-0061] Sun, X. G. , W. Y. Feng , M. Li , J. Shi , and G. J. Ding . 2019. “Phenology and Cultivation of Suillus Bovinus, an Edible Mycorrhizal Fungus, in a *Pinus massoniana* Plantation.” Canadian Journal of Forest Research 48, no. 8: 960–968. 10.1139/cjfr-2018-0405.

[ece371132-bib-0062] Tang, N. , A. Lebreton , W. Xu , Y. Dai , F. Yu , and F. M. Martin . 2021. “Transcriptome Profiling Reveals Differential Gene Expression of Secreted Proteases and Highly Specific Gene Repertoires Involved in Lactarius‐Pinus Symbioses.” Frontiers in Plant Science 12: 714393. 10.3389/fpls.2021.714393. 34490014 PMC8417538

[ece371132-bib-0063] Tedersoo, L. , and M. Bahram . 2019. “Mycorrhizal Types Differ in Ecophysiology and Alter Plant Nutrition and Soil Processes.” Biological Reviews 94, no. 5: 1857–1880. 10.1111/brv.12538. 31270944

[ece371132-bib-0064] Toju, H. , H. Sato , and A. S. Tanabe . 2014. “Diversity and Spatial Structure of Belowground Plant–Fungal Symbiosis in a Mixed Subtropical Forest of Ectomycorrhizal and Arbuscular Mycorrhizal Plants.” PLoS One 9, no. 1: e86566. 10.1371/journal.pone.0086566. 24489745 PMC3904951

[ece371132-bib-0065] Trivedi, P. , J. E. Leach , S. G. Tringe , T. Sa , and B. K. Singh . 2020. “Plant‐Microbiome Interactions: From Community Assembly to Plant Health.” Nature Reviews. Microbiology 18, no. 11: 607–621. 10.1038/s41579-020-0412-1. 32788714

[ece371132-bib-0066] van der Heijden, M. G. A. , R. D. Bardgett , and N. M. van Straalen . 2008. “The Unseen Majority: Soil Microbes as Drivers of Plant Diversity and Productivity in Terrestrial Ecosystems.” Ecology Letters 11: 296–310. 10.1111/j.1461-0248.2007.01139.x. 18047587

[ece371132-bib-0067] Vorholt, J. A. 2012. “Microbial Life in the Phyllosphere.” Nature Reviews. Microbiology 10, no. 12: 828–840. 10.1038/nrmicro2910. 23154261

[ece371132-bib-0068] Walkley, A. , and I. A. Black . 1934. “An Examination of the Degtjareff Method for Determining Soil Organic Matter, and a Proposed Modification of the Chromic Acid Titration Method.” Soil Science 37, no. 1: 29–38.

[ece371132-bib-0069] Wallander, H. , U. Johansson , E. Sterkenburg , M. B. Durling , and B. D. Lindahl . 2010. “Production of Ectomycorrhizal Mycelium Peaks During Canopy Closure in Norway Spruce Forests.” New Phytologist 187, no. 4: 1124–1134. 10.1111/j.1469-8137.2010.03324.x. 20561206

[ece371132-bib-0070] Wang, C. Q. , L. Xue , Y. H. Dong , et al. 2018. “Contrasting Effects of Chinese Fir Plantations of Different Stand Ages on Soil Enzyme Activities and Microbial Communities.” Forests 10, no. 1: 11. 10.3390/f10010011.

[ece371132-bib-0071] Wang, Q. , X. H. He , and L. D. Guo . 2012. “Ectomycorrhizal Fungus Communities of Quercus Liaotungensis Koidz of Different Ages in a Northern China Temperate Forest.” Mycorrhiza 22, no. 6: 461–470. 10.1007/s00572-011-0423-x. 22138969

[ece371132-bib-0072] Wang, Y. Z. , H. F. Zhang , Y. P. Zhang , et al. 2023. “Crop Rotation‐Driven Changes in Rhizosphere Metabolite Profiles Regulate Soil Microbial Diversity and Functional Capacity.” Agriculture, Ecosystems & Environment 358: 108716. 10.1016/j.agee.2023.108716.

[ece371132-bib-0073] Wei, G. F. , K. Ning , G. Z. Zhang , et al. 2021. “Compartment Niche Shapes the Assembly and Network of *Cannabis sativa*‐Associated Microbiome.” Frontiers in Microbiology 12: 714993. 10.3389/fmicb.2021.714993. 34675893 PMC8524047

[ece371132-bib-0074] Wen, H. , K. Xiong , H. Yang , P. Zhang , and X. Wang . 2022. “Dynamic Mechanism of the Microbiota of High‐Salinity Organic Wastewater With Salt‐Tolerant Yeast and Its Application.” Journal of Environmental Chemical Engineering 10, no. 3: 107377. 10.1016/j.jece.2022.107377.

[ece371132-bib-0075] Xin, W. , J. Zhang , Y. Yu , et al. 2024. “Root Microbiota of Tea Plants Regulate Nitrogen Homeostasis and Theanine Synthesis to Influence Tea Quality.” Current Biology 34, no. 4: 868–880. 10.1016/j.cub.2024.01.044. 38366595

[ece371132-bib-0076] Xu, Q. F. , P. K. Jiang , J. S. Wu , G. M. Zhou , R. F. Shen , and J. J. Fuhrmann . 2015. “Bamboo Invasion of Native Broadleaf Forest Modified Soil Microbial Communities and Diversity.” Biological Invasions 17, no. 1: 433–444. 10.1007/s10530-014-0741-y.

[ece371132-bib-0077] Xuan, P. , H. Ma , X. Deng , et al. 2024. “Microbiome‐Mediated Alleviation of Tobacco Replant Problem via Autotoxin Degradation After Long‐Term Continuous Cropping.” iMeta 3, no. 2: e189. 10.1002/imt2.189. 38882490 PMC11170962

[ece371132-bib-0078] Xun, W. B. , Y. P. Liu , W. Li , et al. 2021. “Specialized Metabolic Functions of Keystone Taxa Sustain Soil Microbiome Stability.” Microbiome 9, no. 1: 35. 10.1186/s40168-020-00985-9. 33517892 PMC7849160

[ece371132-bib-0079] Yang, X. , M. Xu , J. Zhang , C. Y. Wen , and J. Zhang . 2023. “Effects of Resin Tapping on Ectomycorrhizal Fungal Community Composition and Structure of *Pinus massoniana* in Subtropical Mountain Forest Ecosystems in Southwestern China.” Forest Ecology and Management 540: 121030. 10.1016/j.foreco.2023.121030.

[ece371132-bib-0080] Yaseen, M. , and S. S. Malhi . 2009. “Differential Growth Performance of 15 Wheat Genotypes for Grain Yield and Phosphorus Uptake on a Low Phosphorus Soil Without and With Applied Phosphorus Fertilizer.” Journal of Plant Nutrition 32, no. 6: 1015–1043. 10.1080/01904160902872818.

[ece371132-bib-0081] Zhang, W. Q. , D. S. Xie , H. F. Yu , J. Deng , and Q. Z. Yao . 2023. “Community Structure and Driving Factors for Rhizosphere Ectomycorrhizal fungi of *Pinus massoniana* L. in Yuntai Mountain.” Bangladesh Journal of Botany 51, no. 40: 913–922. 10.3329/bjb.v51i40.63834.

[ece371132-bib-0082] Zhang, Z. , S. Ge , L. Fan , et al. 2022. “Diversity in Rhizospheric Microbial Communities in Tea Varieties at Different Locations and Tapping Potential Beneficial Microorganisms.” Frontiers in Microbiology 13: 1027444. 10.3389/fmicb.2022.1027444. 36439826 PMC9685800

[ece371132-bib-0083] Zhang, Z. , J. Zhang , and S. Jiao . 2021. “Fungi Show Broader Environmental Thresholds in Wet Than Dry Agricultural Soils With Distinct Biogeographic Patterns.” Science of the Total Environment 750: 141761. 10.1016/j.scitotenv.2020.141761. 32877788

[ece371132-bib-0084] Zhong, Y. , W. Yan , R. Wang , W. Wang , and Z. Shangguan . 2018. “Decreased Occurrence of Carbon Cycle Functions in Microbial Communities Along With Long‐Term Secondary Succession.” Soil Biology and Biochemistry 123: 207–217. 10.1016/j.soilbio.2018.05.017.

[ece371132-bib-0085] Zhou, N. , X. Han , N. Hu , et al. 2024. “The Crop Mined Phosphorus Nutrition via Modifying Root Traits and Rhizosphere micro‐Food Web to Meet the Increased Growth Demand Under Elevated CO2.” iMeta 3, no. 6: e245. 10.1002/imt2.245. 39742301 PMC11683460

[ece371132-bib-0086] Zhu, W. Z. , X. H. Cai , X. L. Liu , et al. 2010. “Soil Microbial Population Dynamics Along a Chronosequence of Moist Evergreen Broad‐Leaved Forest Succession in Southwestern China.” Journal of Mountain Science 7, no. 4: 327–338. 10.1007/s11629-010-1098-z.

