# Supplementary material for: Unveiling Microbial Dynamics: How Forest Aging Shapes the Microbial Communities of Pinus massoniana
Source: Ecol Evol. 2025 Mar 11;15(3):e71132. doi: 10.1002/ece3.71132 (PMC11896641; doi:10.1002/ece3.71132)
Supplement: Supplementary file 1 — Figure S1. [file ECE3-15-e71132-s001.zip › ece371132-sup-0001-Revised Supplementary material-figures0303.docx]

***Supporting information for***

**Unveiling Microbial Dynamics: How Forest Aging Shapes the Microbial Communities of *Pinus massoniana***

Guiyun Yuan^1,2,3^, Yang Zheng^1,2,3^, Xueguang Sun^1,2,3^ *

^1^ Institute for Forest Resources & Environment of Guizhou, Guizhou University, Guiyang 550025, China

^2^ Key Laboratory of Forest Cultivation in Plateau Mountain of Guizhou Province, Guizhou University, Guiyang, Guizhou, 550025, China

^3^ College of Forestry, Guizhou University, Guiyang 550025, China

***Corresponding author: Xueguang Sun

Address: Institute for Forest Resources & Environment of Guizhou, Guizhou University, Guiyang 550025, China

E-mail: sunxg0518@aliyun.com

Tel：+86 18302544760


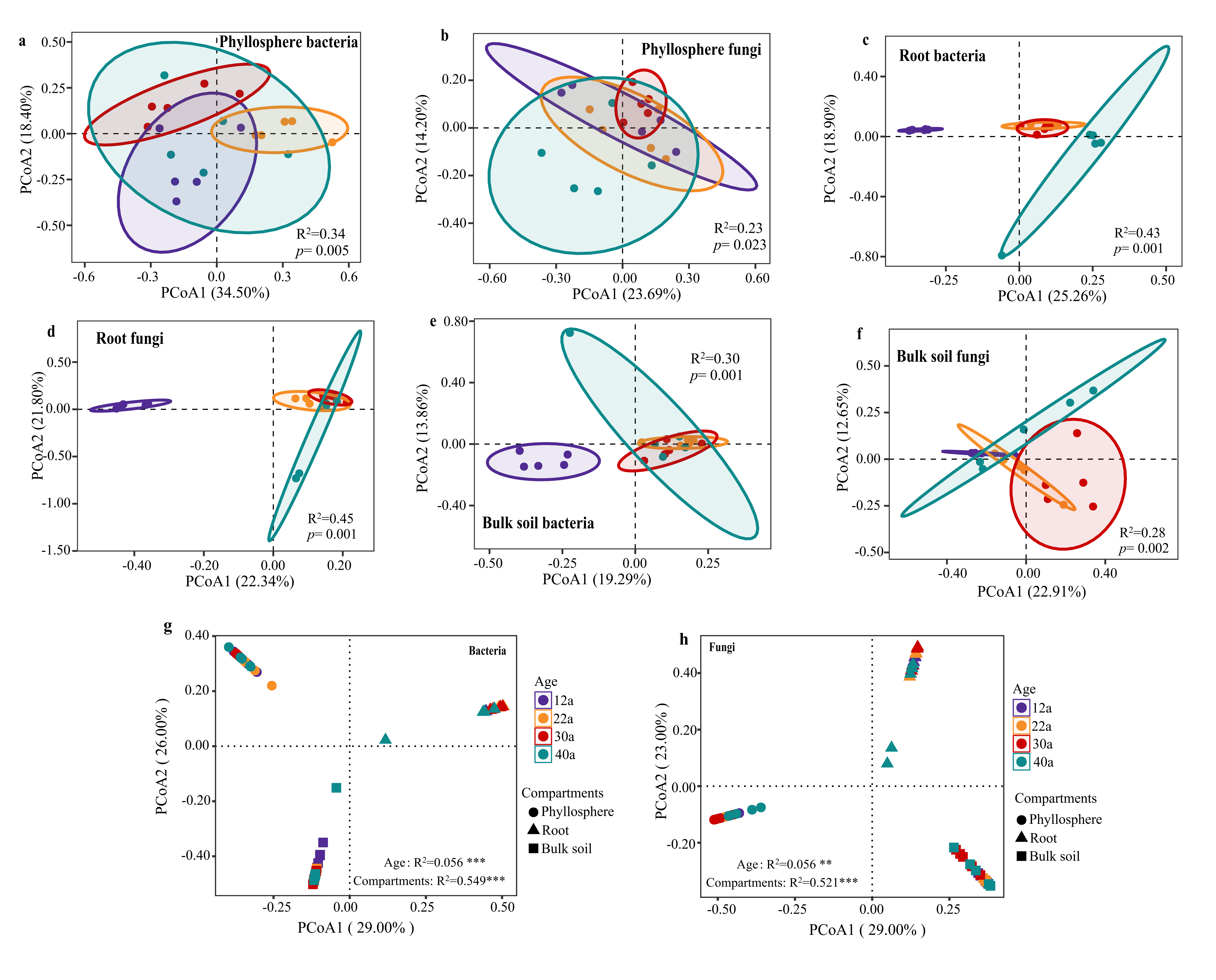


**Figure S1.** Principal coordinates analysis (PCoA) of microbial communities based on Bray-Curtis distance. a-f indicate the principal component analysis of microorganisms in different forest ages, and g-h compare and analyze the effects of ecological niche and forest age on bacterial and fungal communities. ****P*<0.001, **0.001<*P<*0.01.

**
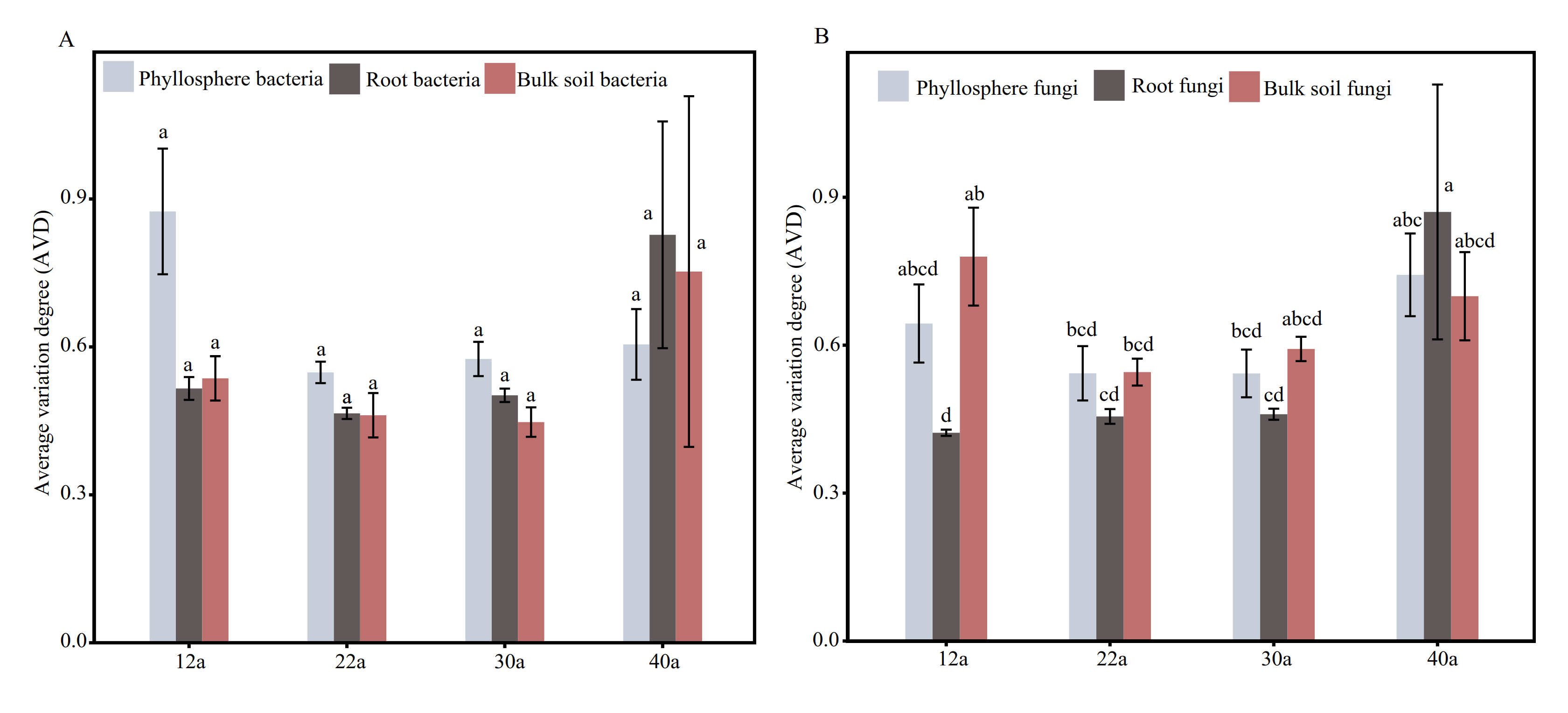
Figure S2.** AVD of microorganisms at different forest ages. Different letters on the bars indicate significant differences in the average variability among different groups of microorganisms, *P <* 0.05.


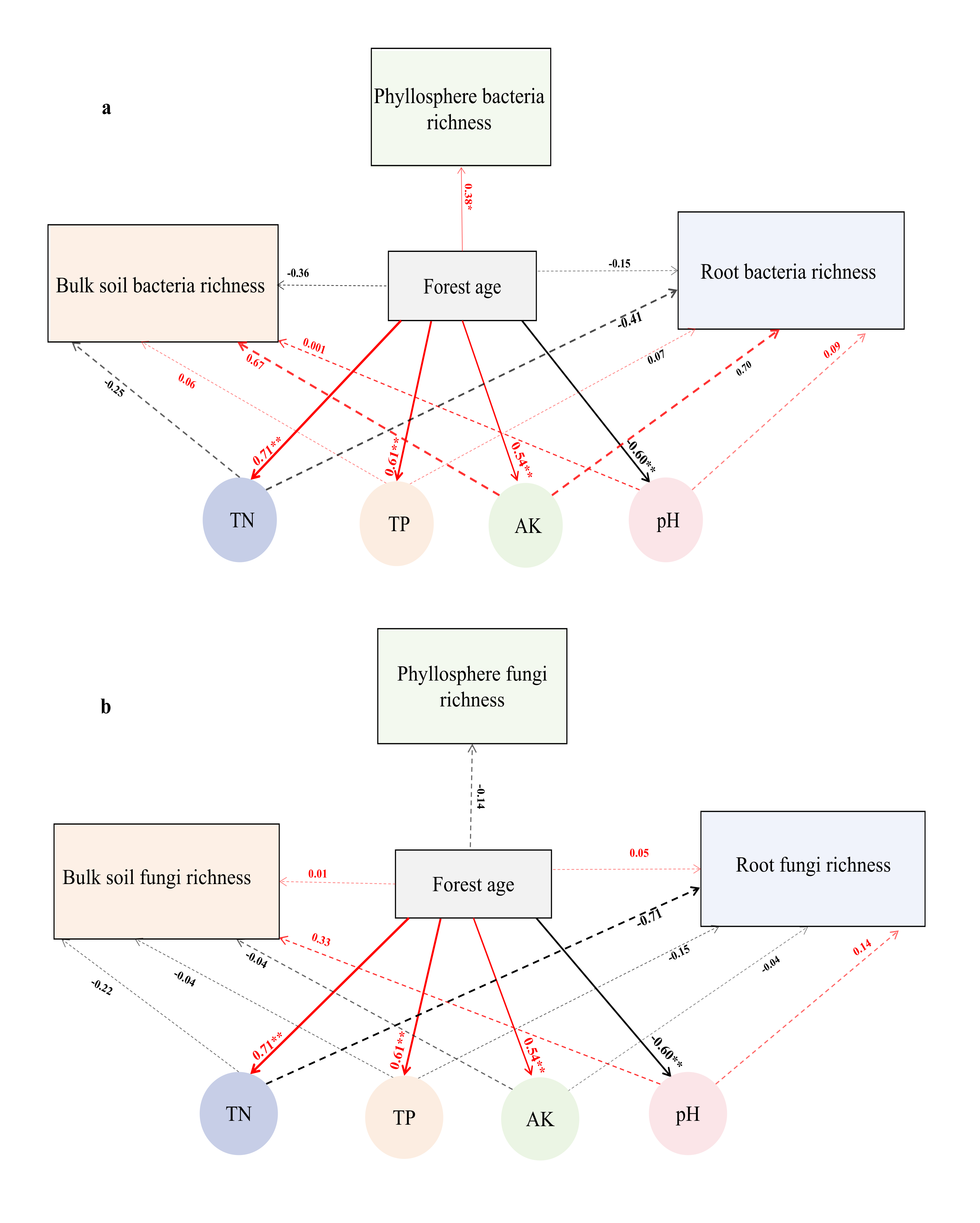


**Figure S3.** Effects of different environmental factors on the microbial diversity of *P. massoniana.* a refers to the SEM model of bacterial communities of *P. massoniana*; b refers to the SEM model of fungi communities of *P. massoniana*; **P*<0.05, ***P*<0.01. The red line shows positive correlation, the black line shows negative correlation, the solid line shows *P*<0.05, the dotted line shows *P*≥0.05, if the line is thicker and clearer, it means that the correlation between the indicators is stronger.

**Comparison of core microorganisms in different ecological niches of *P. massoniana***

Zi-Pi plots were constructed to confirm the topological roles of bacterial and fungal network nodes (Fig. S4). Nodes assigned to module hub, network hub, and connectors were considered as key species in the microbial community (Montoya et al., 2006). We observed different numbers of connectors in the phyllosphere, root and soil microorganisms, with root bacteria having the largest number of connectors (72 nodes, Fig. S4c) and phyllosphere fungi having the smallest number of connectors (6 nodes, Fig. S4b). And module hubs only exist in co-occurrence networks of root bacteria (4 nodes) and soil bacteria (4 nodes), which were highly connected to many nodes in their modules. After statistics, we found that 45 nodes, 72 nodes and 46 nodes in the phyllosphere bacterial network, root bacterial network and soil bacterial network were classified as connectors, respectively (Fig. S4a, c, e). And there were 6 nodes, 17 nodes and 22 nodes in phyllosphere, root and soil fungal network respectively categorized as connectors (Fig. S4b, d, f).


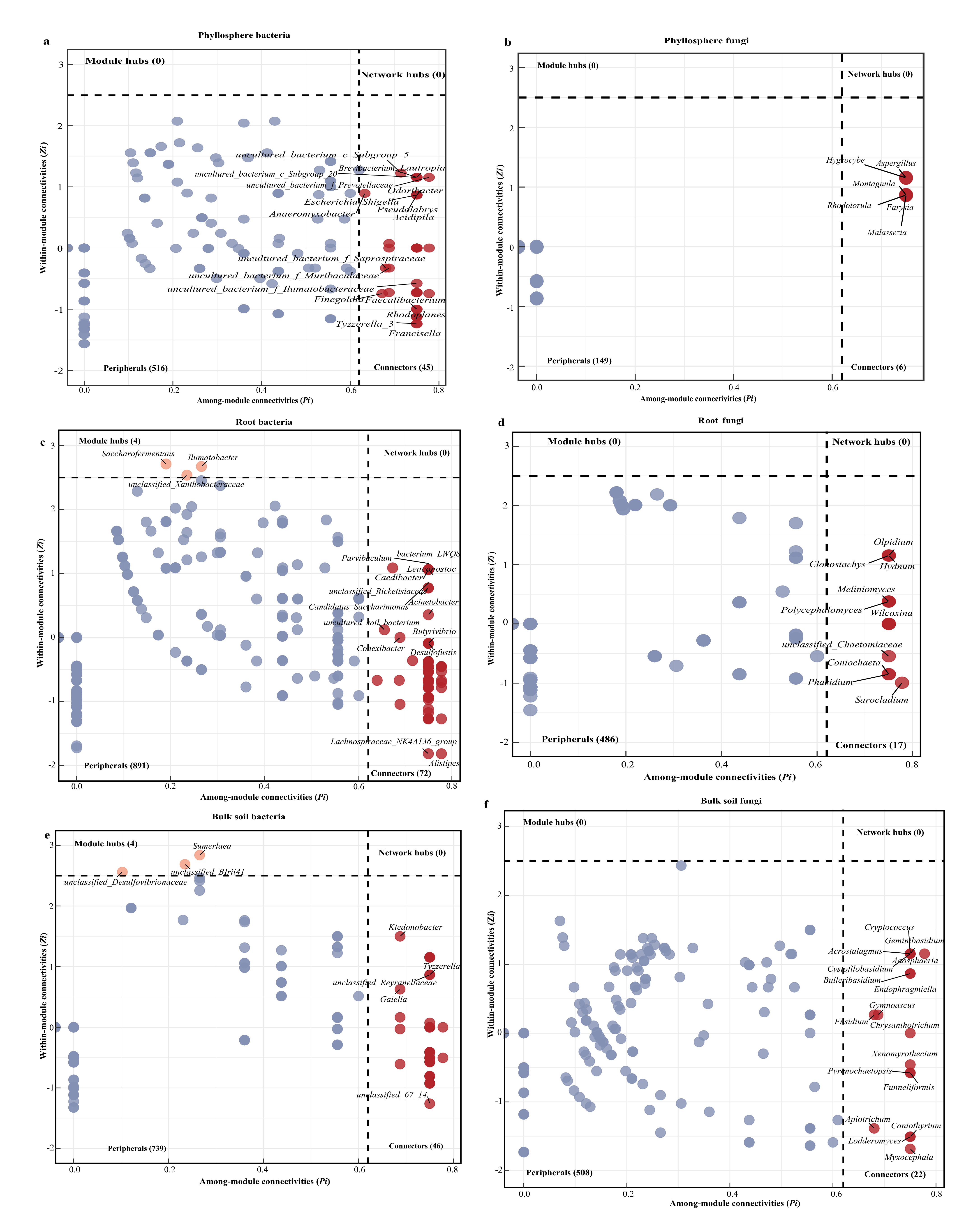


**Figure S4.** Zi-Pi plots of bacterial and fungal based on ASVs topological roles in co-occurrence network of microbial communities. The number in brackets indicates the number of ASVs belonging to the corresponding module.

**Correlation between soil microbial communities and soil physicochemical properties**

In order to explore the driving factors of soil microbial differences among different forest ages of *P. massoniana*, we conducted a correlation analysis between the soil microbial richness of different forest ages and eight soil physical and chemical indicators. The results found that total nitrogen (R^2^=0.25, *P*<0.05), Total phosphorus (R^2^=0.22, *P*<0.05) has a significant negative linear relationship with the species richness of the soil fungal community, and pH (R^2^=0.27, *P*<0.05) has a significant positive linear relationship with the species richness of the soil fungal community (Fig. S5b). However, none of the eight soil physical and chemical indicators significantly affected soil bacterial richness (Fig. S5a).


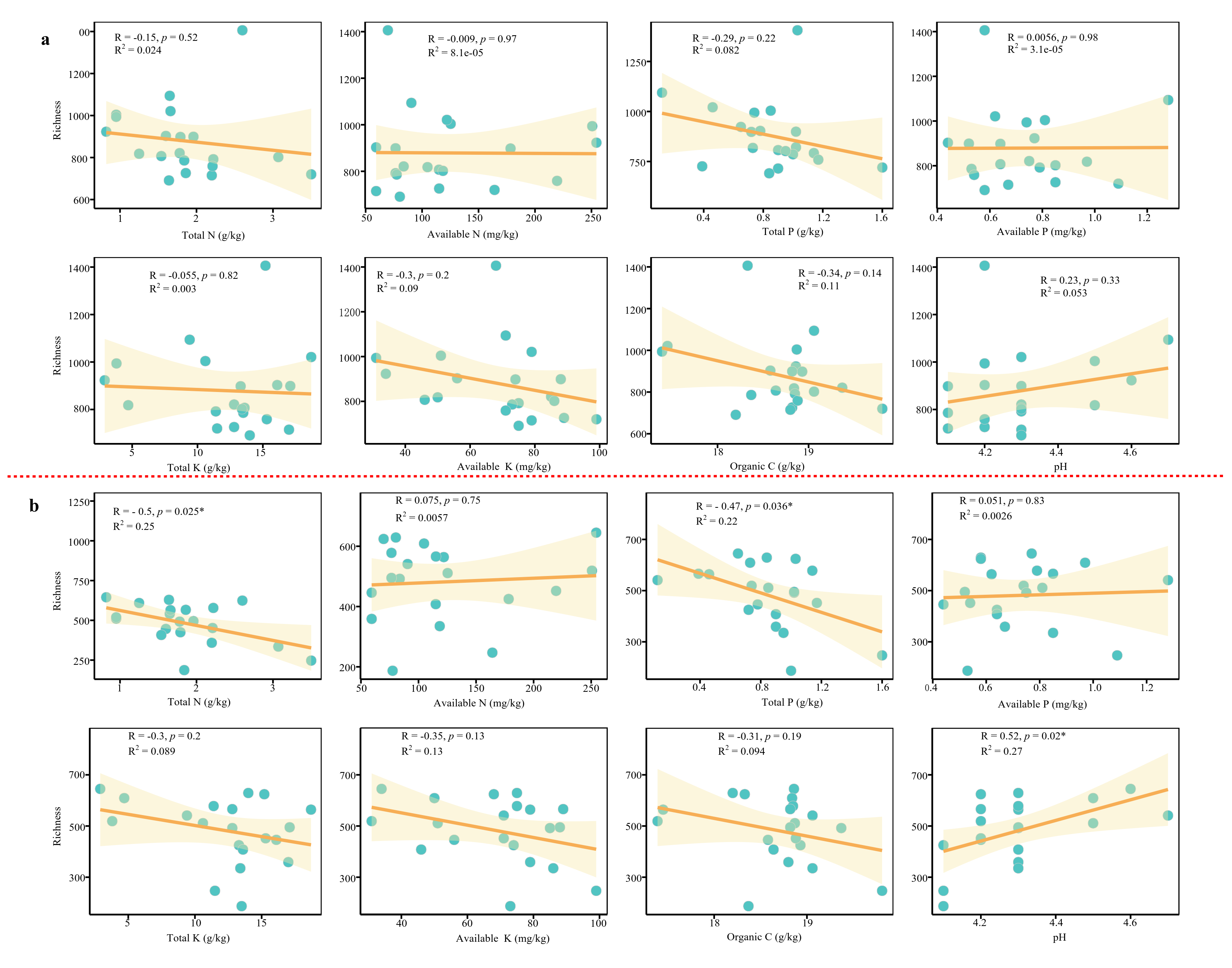


**Figure S5.** Linear regression analysis of soil physical and chemical properties and soil microbial community species richness. a refers to soil bacteria, b refers to soil fungi. *indicates that the richness of microbial communities is significantly related to the corresponding physical and chemical properties (*P*<0.05).

**References**

Montoya, J.M., Pimm, S.L., Solé, R.V., 2006. Ecological networks and their fragility. Nature 442(7100), 259-264. https://doi.org/10.1038/nature04927
