# Supplementary material for: Unveiling Microbial Dynamics: How Forest Aging Shapes the Microbial Communities of Pinus massoniana
Source: Ecol Evol. 2025 Mar 11;15(3):e71132. doi: 10.1002/ece3.71132 (PMC11896641; doi:10.1002/ece3.71132)
Supplement: Supplementary file 2 — Table S1: [file ECE3-15-e71132-s002.docx]

**Table S1. Physicochemical properties of different pine forest soils**

| Sample sites | pH | Total nitrogen  (g/kg) | Alkaline nitrogen  (mg/kg) | Total phosphorus  (g/kg) | Available phosphorus  (mg/kg) | Total potassium  (g/kg) | Available potassium  (mg/kg) | Organic matter  (g/kg) |
| --- | --- | --- | --- | --- | --- | --- | --- | --- |
| 12a | 4.50±0.08a | 1.12±0.15c | 165.14±36.11a | 0.62±0.13b | 0.91±0.10a | 6.28±1.56b | 47.40±7.16b | 18.60±0.31a |
| 22a | 4.24±0.04b | 1.86±0.10b | 115.13±23.31ab | 0.75±0.15ab | 0.73±0.04ab | 13.80±1.27a | 80.40±2.89a | 18.68±0.32a |
| 30a | 4.24±0.04b | 1.83±0.12b | 77.53±13.12b | 0.92±0.42ab | 0.56±0.04b | 15.46±0.80a | 68.40±7.66ab | 18.64±0.08a |
| 40a | 4.22±0.04b | 2.60±0.32a | 130.43±27.77ab | 1.12±0.13a | 0.73±0.11ab | 13.88±0.70a | 79.80±5.69a | 18.86±0.29a |

Note: Different letters after the same column of data represent significant differences between the data (*P* < 0.05).
